# Depletion of Retinal Dopaminergic Activity in a Mouse Model of Rod Dysfunction Exacerbates Experimental Autoimmune Uveoretinitis: A Role for the Gateway Reflex

**DOI:** 10.3390/ijms23010453

**Published:** 2021-12-31

**Authors:** Andrea Stofkova, Miloslav Zloh, Dominika Andreanska, Ivana Fiserova, Jan Kubovciak, Jan Hejda, Patrik Kutilek, Masaaki Murakami

**Affiliations:** 1 Department of Physiology, Third Faculty of Medicine, Charles University, Ke Karlovu 4, 120 00 Prague, Czech Republic; miloslav.zloh@lf3.cuni.cz (M.Z.); andreanska.dominika@gmail.com (D.A.); ivana.fiserova@lf3.cuni.cz (I.F.); 2 Department of Pathophysiology, Third Faculty of Medicine, Charles University, Ruska 87, 100 00 Prague, Czech Republic; 3 Laboratory of Genomics and Bioinformatics, Institute of Molecular Genetics of the Czech Academy of Sciences, Videnska 1083, 142 20 Prague, Czech Republic; jan.kubovciak@img.cas.cz; 4 Department of Health Care and Population Protection, Faculty of Biomedical Engineering, Czech Technical University in Prague, Sitna Sq. 3105, 272 01 Kladno, Czech Republic; jan.hejda@fbmi.cvut.cz (J.H.); kutilek@fbmi.cvut.cz (P.K.); 5 Division of Molecular Psychoimmunology, Institute for Genetic Medicine and Graduate School of Medicine, Hokkaido University, Kita-15, Nishi-7, Kita-ku, Sapporo 060-0815, Japan; murakami@igm.hokudai.ac.jp

**Keywords:** experimental autoimmune uveoretinitis, gateway reflex, dopamine, Gnat1, night blindness, rod-cone dystrophy, blood–retinal barrier, endothelial cells, NF-κB, STAT3

## Abstract

The gateway reflex is a mechanism by which neural inputs regulate chemokine expression at endothelial cell barriers, thereby establishing gateways for the invasion of autoreactive T cells into barrier-protected tissues. In this study, we hypothesized that rod photoreceptor dysfunction causes remodeling of retinal neural activity, which influences the blood–retinal barrier and the development of retinal inflammation. We evaluated this hypothesis using *Gnat1^rd17^* mice, a model of night blindness with late-onset rod-cone dystrophy, and experimental autoimmune uveoretinitis (EAU). Retinal remodeling and its effect on EAU development were investigated by transcriptome profiling, target identification, and functional validation. We showed that *Gnat1^rd17^* mice primarily underwent alterations in their retinal dopaminergic system, triggering the development of an exacerbated EAU, which was counteracted by dopamine replacement with L-DOPA administered either systemically or locally. Remarkably, dopamine acted on retinal endothelial cells to inhibit NF-κB and STAT3 activity and the expression of downstream target genes such as chemokines involved in T cell recruitment. These results suggest that rod-mediated dopamine release functions in a gateway reflex manner in the homeostatic control of immune cell entry into the retina, and the loss of retinal dopaminergic activity in conditions associated with rod dysfunction increases the susceptibility to autoimmune uveitis.

## 1. Introduction

Autoimmune uveitis (AU) is an intraocular inflammatory disease that primarily affects adults in their most active and productive years [1]. AU is mostly an idiopathic condition [2] that is believed to develop as a result of the activation and expansion of ocular antigen-specific CD4^+^ T helper cells accumulating in the eye [3]. Depending on the anatomical location of the inflammatory pathology, AU is categorized into four types: (1) anterior uveitis, manifesting as iritis, iridocyclitis, and anterior cyclitis; (2) intermediate uveitis, manifesting as pars planitis, posterior cyclitis, and hyalitis; (3) posterior uveitis presenting as focal, multifocal, or diffuse choroiditis, chorioretinitis, retinitis, and diffuse neuroretinitis; and (4) panuveitis involving diffuse inflammation of many areas of the eye, such as the anterior chamber, vitreous, retina, and choroid [2]. Inflammation affecting the posterior segment of the eye is frequently associated with a substantial risk of ocular complications, permanent visual impairment, or even blindness [4,5]. Furthermore, it has been reported that some forms of AU, including birdshot retinochoroidopathy and sarcoid chorioretinopathy, exhibit night blindness, a typical feature of retinitis pigmentosa (RP) [6,7,8,9]. RP refers to a heterogenic group of inherited retinal diseases characterized by a progressive degeneration of rod and cone photoreceptors. In most cases, RP is described as rod–cone dystrophy due to primary degeneration of rods that precedes the cone loss. It is initially manifested by symptoms of night blindness, followed by peripheral visual field constriction and by loss of central vision in the most advanced stages [10]. Several studies have also demonstrated that uveitis is not an uncommon condition in RP patients [11,12,13,14]. However, the underlying mechanism explaining why some patients with RP are prone to AU, and vice versa, is largely unknown and remains an important issue to be addressed. 

The breakdown of the blood–retinal barrier (BRB) plays an essential role in the pathogenesis of posterior AU [15]. In RP, it has also been shown that the majority of patients, including those with no other ocular disorders, exhibited extravascular albumin in the inner portion of the posterior retina [16]. This indicates that the BRB breakdown might be an overlapping pathological feature in both RP and AU. The retinal endothelium with a lack of fenestrations and the presence of specialized tight junctions is a crucial component of the BRB [15]. Vascular endothelial cells respond to various signals and stimuli, including hormones, cytokines, and neurotransmitters [17]. We have previously reported that certain sensory neural inputs can be converted either to pro-inflammatory or anti-inflammatory signals for endothelial cells of the blood–brain barrier (BBB) and the BRB to control an establishment of gateways for autoreactive CD4^+^ T-cell infiltration into the neural tissue [18,19,20,21]. This neurogenic mechanism is termed the gateway reflex, and its molecular basis is the inflammation amplifier, a positive feedback loop between pro-inflammatory cytokines IL-6 and IL-17, and transcription factors STAT3 and NF-κB. The inflammation amplifier maintains a persistent and massive expression of a variety of chemokines, cytokines, and growth factors by non-immune cells like endothelial cells, thereby promoting recruitment of immune cells in affected tissues [22,23,24,25,26,27]. Importantly, the ability of the photopic light-mediated neural activity (the light gateway reflex) to suppress the inflammation amplifier in retinal endothelial cells reduced immune cell entry in the retina and the progression of experimental autoimmune uveoretinitis (EAU) [21], a murine model of autoimmune posterior uveitis/panuveitis [28,29].

In this study, we aimed to elucidate whether rod dysfunction, a typical sign of early RP, creates conditions that increase the severity of EAU induced by active immunization with retinal antigen interphotoreceptor retinoid-binding protein (IRBP) and which mechanism may be the cause. Based on our previous findings on the light gateway reflex [21], we hypothesized that rod dysfunction might disrupt homeostatic neural network activity in the retina capable of controlling gateways for immune cell trafficking across the BRB to the retina in the EAU model.

We tested this hypothesis using *Gnat1^rd17^* mice, a model of naturally occurring autosomal recessive rod dysfunction rd17 [30,31,32,33]. *Gnat1^rd17^* mice are homozygous for the nonsense *Gnat1^irdr^* mutation and resemble the phenotype of IRD2 mice that also carry the *Gnat1^irdr^* allele, resulting in nonfunctional truncated protein product Gnat1 (rod transducin α subunit), a key player in the rod phototransduction pathway [33,34,35]. These mice exhibit early night blindness and progressive reduction in rod photoreceptors beginning at 6 months of age, which makes them a suitable model of human congenital stationary night blindness (CSNB) associated with *Gnat1* mutation and late rod–cone dystrophy [33,36,37,38].

## 2. Results

### 2.1. Gnat1^rd17^ Mice Exhibit Aggravated EAU Severity 

**Figure 1:**
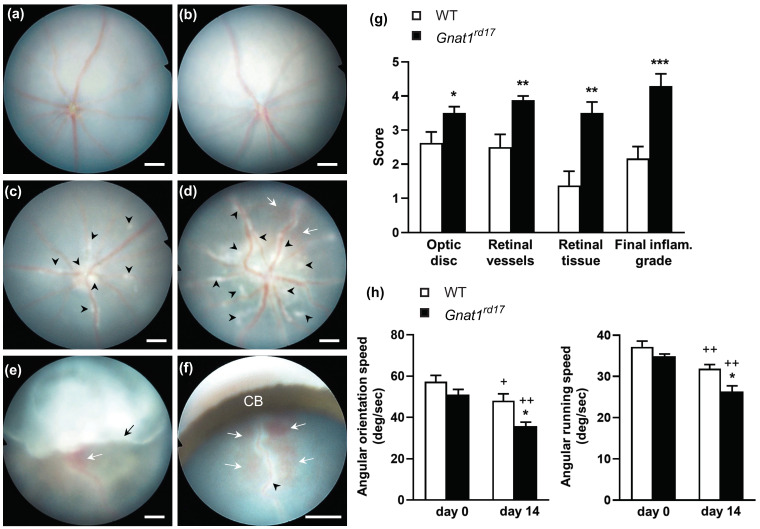
*Gnat1^rd17^* mice exhibit exacerbated EAU. Fundus images of normal 9-week-old (**a**) WT and (**b**) *Gnat1^rd17^* mice before EAU induction. Fundus images of EAU (**c**) WT and (**d**–**f**) *Gnat1^rd17^* mice taken on day 14 after immunization. *Gnat1^rd17^* mice with EAU showed signs of hemorrhage (white arrows) and more inflammatory infiltrates (black arrowheads) in the retina than EAU WT mice. (**e**) Fundus image of EAU *Gnat1^rd17^* mouse with severe retinal detachment (black arrow) on day 14 after immunization. (**f**) Magnification of the peripheral retina shows vessel hemorrhage and severe perivascular inflammatory infiltrates observed in EAU *Gnat1^rd17^* mice on day 14 after immunization. CB, ciliary body. (**g**) Results of EAU clinical score evaluated by fundus examination in EAU WT and *Gnat1^rd17^* mice on day 14 after immunization. Data are expressed as mean ± SEM of 10 eyes from 5 mice per group; one representative out of three experiments is shown. Differences between groups: * *p* 0.05, ** *p* 0.01, and *** *p* 0.001 (unpaired *t*-test). (**h**) Optomotor responses, angular orientation speed, and angular running speed, measured in the optomotor drum in WT and *Gnat1^rd17^* mice shortly before immunization (day 0) and on day 14 after immunization. Data shown are mean ± SEM of 5 mice in each group; one representative out of two experiments is shown. ^+^ *p* 0.05 and ^++^ *p* 0.01 by paired *t*-test for comparisons between day 0 and day 14 within the same group and * *p* 0.05 by unpaired *t*-test for comparisons between WT and *Gnat1^rd17^* mice. Scale bar: 200 μm.

To investigate the clinical expression of EAU in *Gnat1^rd17^* mice that are deficient in rod phototransduction, we induced EAU by active immunization with uveitogenic antigen and human IRBP_1–20_ peptide in *Gnat1^rd17^* and wild-type (WT) mice. At the time of immunization (8 to 10 weeks of age), fundus examination did not show any signs of retinal or vascular pathological changes in either *Gnat1^rd17^* or WT mice (Figure 1a,b), which is consistent with previous findings [30,33]. In the central and peripheral retina, no significant differences in the thickness of the outer nuclear layer (ONL) or inner nuclear layer (INL) were observed in 8- to 10-week-old *Gnat1^rd17^* mice compared to age-matched WT mice (Figure S5). Because the ONL is composed of photoreceptors, these findings indicate that photoreceptor degeneration is not present in *Gnat1^rd17^* mice at the age of EAU induction. Likewise, the visual acuity test, as determined by the optomotor response test, a commonly used visual acuity screening method for mice that measures head and/or body movements in response to moving black and white vertical rotating stripes across the visual field [39,40], showed no impairment of visual function in *Gnat1^rd17^* mice compared to WT mice on day 0, shortly before the immunization (Figure 1h).

When we evaluated the development of EAU in *Gnat1^rd17^* and WT mice, we observed that the first clinical signs of EAU appeared on the fundus around day 10 to 12 following immunization, which is a typical onset of EAU in C57BL/6 mice [21,28,41]. Although no differences in disease score were found for the initiation of EAU (data not shown), the differences between *Gnat1^rd17^* and WT mice in the EAU clinical outcome from day 14 after immunization were significant (Figure 1g). In *Gnat1^rd17^* mice, we observed more severe retinal inflammation, characterized by the presence of extensive inflammatory infiltrates around retinal vessels (perivascular cuffing), linear lesions, retinal detachments, and hemorrhage (Figure 1d–f) compared to WT mice (Figure 1c). In some *Gnat1^rd17^* mice, we observed a large retinal detachment that obstructed the view of the optic disc (Figure 1f), which we did not find in the fundus of WT mice on day 14 after immunization. Furthermore, the results of visual acuity testing using the optomotor response method demonstrated a significant decrease in the angular orientation speed and angular running speed in EAU *Gnat1^rd17^* mice compared to WT mice on day 14 after immunization (Figure 1h). These data indicate an exacerbation of EAU and a more pronounced deterioration in visual function in EAU *Gnat1^rd17^* mice than in their WT controls. 

### 2.2. Exacerbation of EAU in Gnat1^rd17^ Mice Is Associated with Abundant Immune-Cell Infiltrates and Retinal Neuroinflammatory Response 

**Figure 2:**
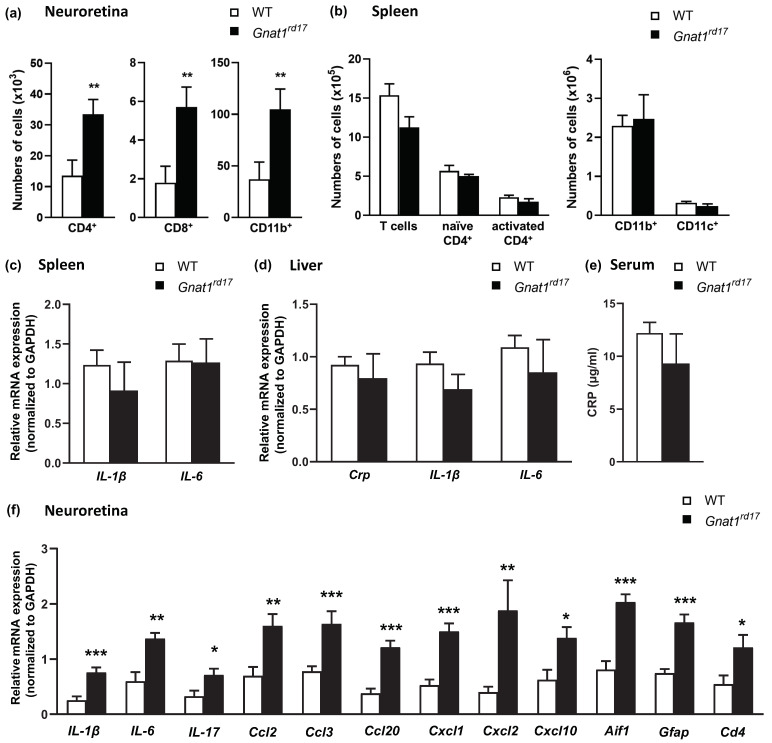
EAU in *Gnat1^rd17^* mice results in an increased accumulation of immune cells in the neuroretina accompanied by local overexpression of inflammatory mediators. (**a**) Quantification of total CD4^+^, CD8^+^, and CD11b^+^ cell infiltrates in the neuroretina by flow cytometry in EAU WT and *Gnat1^rd17^* mice on day 14 after immunization. Data are given as the mean ± SEM of 10 neuroretinas per group. Differences between groups: ** *p* 0.01 by unpaired *t*-test. (**b**) Flow cytometry analysis of splenocytes collected from EAU WT and *Gnat1^rd17^* mice on day 14 after immunization shows no statistically significant difference between the groups for total numbers of CD3^+^ T cells, naïve CD4^+^ T cells (CD4^+^CD44^low^), activated CD4^+^ T cells (CD4^+^CD44^high^), CD11b^+^ (CD11b^high^CD11c^low^, macrophages, and neutrophils), or CD11c^+^ (CD11c^high^MHC-II^high^, dendritic cells). Data are shown as mean ± SEM of 5 mice in each group (unpair *t*-test). (**c**) Spleen mRNA expression of *IL-1β* and *IL-6*; (**d**) liver mRNA expression of *Crp*, *IL-1β*, and *IL-6*; and (**e**) serum CRP levels show no statistically significant difference between WT and *Gnat1^rd17^* mice on day 14 after immunization. Data shown are the mean ± SEM of 5 mice in each group (unpaired *t*-test). (**f**) mRNA levels of cytokine, chemokine, and neuroinflammatory/immune cell marker genes in the neuroretinas of EAU WT and *Gnat1^rd17^* mice on day 14 after immunization. The data are presented as the mean ± SEM of 8–10 neuroretinas from 4–5 mice per group, with one representative of two experiments shown. Significant differences between groups: * *p* 0.05, ** *p* 0.01, and *** *p* 0.001 (unpaired *t*-test).

The initial acute phase of EAU (14 days after immunization) also revealed increased absolute numbers of CD4^+^ T cells, CD8^+^ T cells, and CD11b^+^ myeloid cells in the neuroretinas of *Gnat1^rd17^* mice compared to WT mice, as determined by flow cytometry (Figure 2a). The increased infiltrates of CD4^+^ T cells and CD11b^+^ myeloid cells in the retina of EAU *Gnat1^rd17^* mice versus WT mice were also confirmed by immunohistochemistry, as shown in Figure S2.

To compare the immune-cell infiltration with neuroinflammatory alterations in the retina, we examined the gene expression of several mediators of inflammation. We found upregulated expression of genes encoding crucial cytokines that promote EAU development, such as IL-6, IL-17A, and IL-1β, and chemokines, which play a pivotal role in the migration of T cells and other immune cells through the BRB, in the neuroretina of *Gnat1^rd17^* mice compared to WT mice (Figure 2f). In addition, *Aif1* (a marker of activated microglia/macrophages) and *Gfap* (a marker of astrogliosis) mRNA expressions were approximately two-fold higher in the neuroretinas of EAU *Gnat1^rd17^* mice compared with their expressions in the neuroretinas of EAU WT mice (Figure 2f). Given that systemic inflammation associated with elevated circulating C-reactive protein (CRP) levels can contribute to the development of retinal diseases, including uveitis and retinal degeneration [42,43], we next examined whether EAU *Gnat1^rd17^* mice exhibit a higher degree of systemic inflammation than EAU WT mice. We observed that serum concentration and liver mRNA expression of CRP did not differ in EAU *Gnat1^rd17^* mice compared to EAU WT mice on day 14 after immunization (Figure 2d,e). Consistently, there were no differences in liver mRNA expression of genes encoding pro-inflammatory cytokines IL-6 and IL-1β, which are known to stimulate CRP production [44], between EAU *Gnat1^rd17^* and EAU WT mice (Figure 2d). In addition, comparable absolute numbers of splenic naïve and activated CD4^+^ T cells, CD11b^+^ cells (macrophage and neutrophils), and CD11c^+^ dendritic cells, as well as mRNA expressions for IL-6 and IL-1β in the spleen, suggest that both groups of EAU mice developed a similar degree of systemic inflammation on day 14 after immunization (Figure 2b,c). Overall, these findings demonstrate that exacerbation of EAU in *Gnat1^rd17^* mice is not mediated by a systemic inflammatory process but by local neuroinflammatory changes within the retina.

### 2.3. Transcriptomic Changes in the Neuroretina of Gnat1^rd17^ Mice Revealed Deregulation of DA- and Inflammation-Associated Pathways

To elucidate why *Gnat1^rd17^* mice are susceptible to the development of more serious EAU despite showing no abnormalities on the fundus examination or in the optomotor response before immunization, we profiled the neuroretina of *Gnat1^rd17^* mice to characterize transcriptional features unique to this phenotype. Transcriptome profiles were generated by RNA-seq of neuroretinas from healthy *Gnat1^rd17^* and WT mice at the age of 10 weeks. 

**Figure 3:**
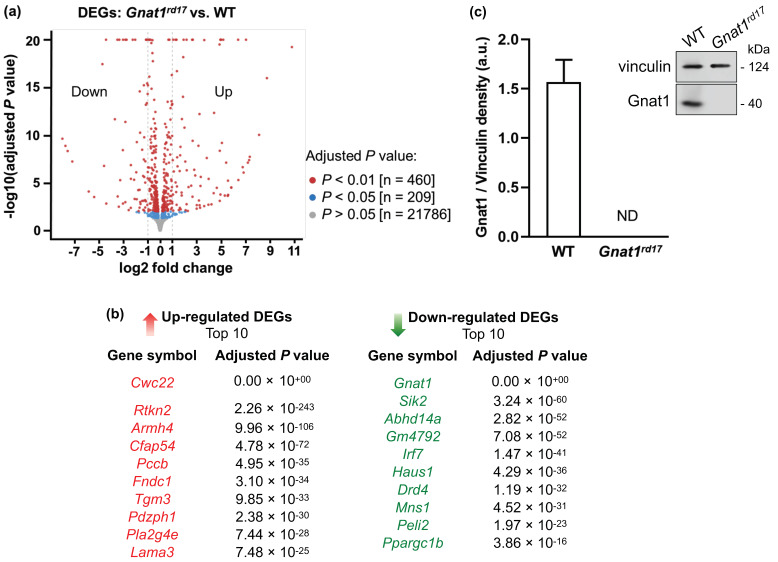
Retinal transcriptomic remodeling in *Gnat1^rd17^* mice. (**a**) Volcano plot showing DEGs in the neuroretina of *Gnat1^rd17^* mice compared to WT mice. The vertical dot lines define upregulated (log2 fold change 1) and downregulated (log2 fold change −1) DEGs. The red and blue dots indicate all genes at adjusted *p* 0.01 and *p* 0.05, respectively. The grey dots represent all genes with an adjusted *p* 0.05. (**b**) The top 10 most significantly upregulated and downregulated protein-coding DEGs, as determined by adjusted *p*-value. (**c**) Representative Western blot images and relative protein expression of Gnat1 assessed by densitometry. Vinculin was used as a protein-loading control. The data represent the mean ± SEM of 5 neuroretinas of five normal WT and five *Gnat1^rd17^* mice at the age of 10 weeks. The data shown are representative of three independent experiments. ND, not detected. Abbreviations: *Cwc22*—CWC22 spliceosome-associated protein; *Rtkn2*—rhotekin 2; *Armh4*—armadillo-like helical domain-containing 4; *Cfap54*—cilia and flagella-associated protein 54; *Pccb*—propionyl Coenzyme A carboxylase, beta polypeptide; *Fndc1*—fibronectin type III domain-containing 1; *Tgm3*—transglutaminase 3, E polypeptide; *Pdzph1*—PDZ and pleckstrin homology domains 1; *Pla2g4e*—phospholipase A2, group IVE; *Lama3*—laminin, alpha 3; *Gnat1*—guanine nucleotide-binding protein, alpha transducing 1; *Sik2*—salt inducible kinase 2; *Abhd14a*—abhydrolase domain-containing 14A; *Gm4792*—predicted gene 4792; *Irf7*—interferon regulatory factor 7; *Haus1*—HAUS augmin-like complex, subunit 1; *Drd4*—DA receptor D4; *Mns1*—meiosis-specific nuclear structural protein 1; *Peli2*—pellino 2; *Ppargc1b*—peroxisome proliferative activated receptor, gamma, coactivator 1 beta.

The comparison of transcriptome profiles of *Gnat1^rd17^* and WT mice revealed 166 differentially expressed genes (DEGs) in the neuroretinas of *Gnat1^rd17^* mice, containing 95 upregulated and 71 downregulated genes (Figure 3a and Table S1). Among the upregulated DEGs, 32 were protein-coding genes, 11 were lncRNAs, 48 were pseudogenes, 1 was a ribozyme, and 3 were transcription elongation complexes (TECs). The downregulated DEGs included 43 protein-coding genes, 14 lncRNAs, 6 pseudogenes, and 8 TECs. As expected, the *Gnat1* gene was the most downregulated gene in the neuroretina of *Gnat1^rd17^* mice (Figure 3b). The absence of a normal (full-length) protein expression was also observed for the *Gnat1* gene product in the neuroretina of *Gnat1^rd17^* mice by Western blot analysis (Figure 3c and Figure S4a), which agrees with the Jackson Laboratory specification of the *Gnat1^rd17^* phenotype [30,33]. The most upregulated gene in the neuroretina of *Gnat1^rd17^* mice, *Cwc20*, was a CWC22 spliceosome-associated protein (Figure 3b) that functions as the major partner of CWC27 (CWC27 spliceosome-associated cyclophilin), a splicing factor linked to retinal degeneration and other developmental defects [45,46].

**Figure 4:**
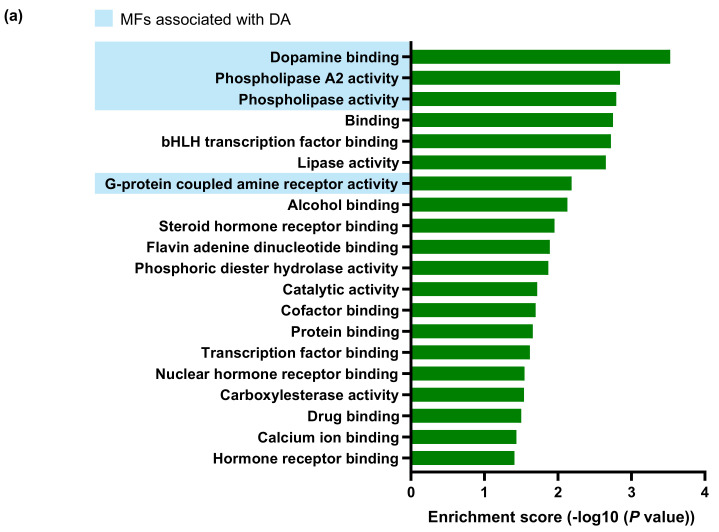

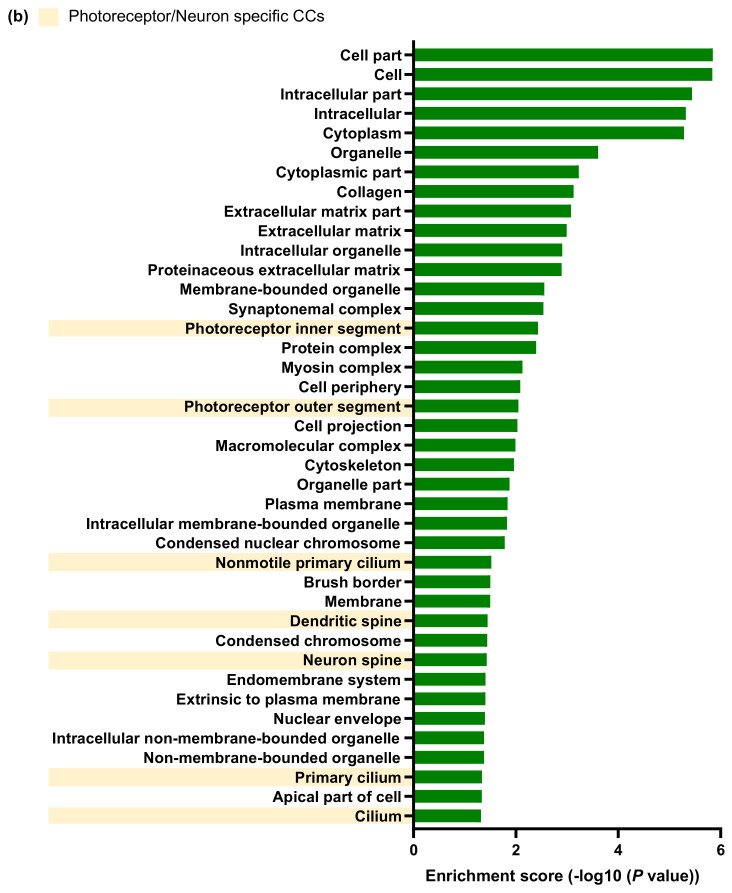

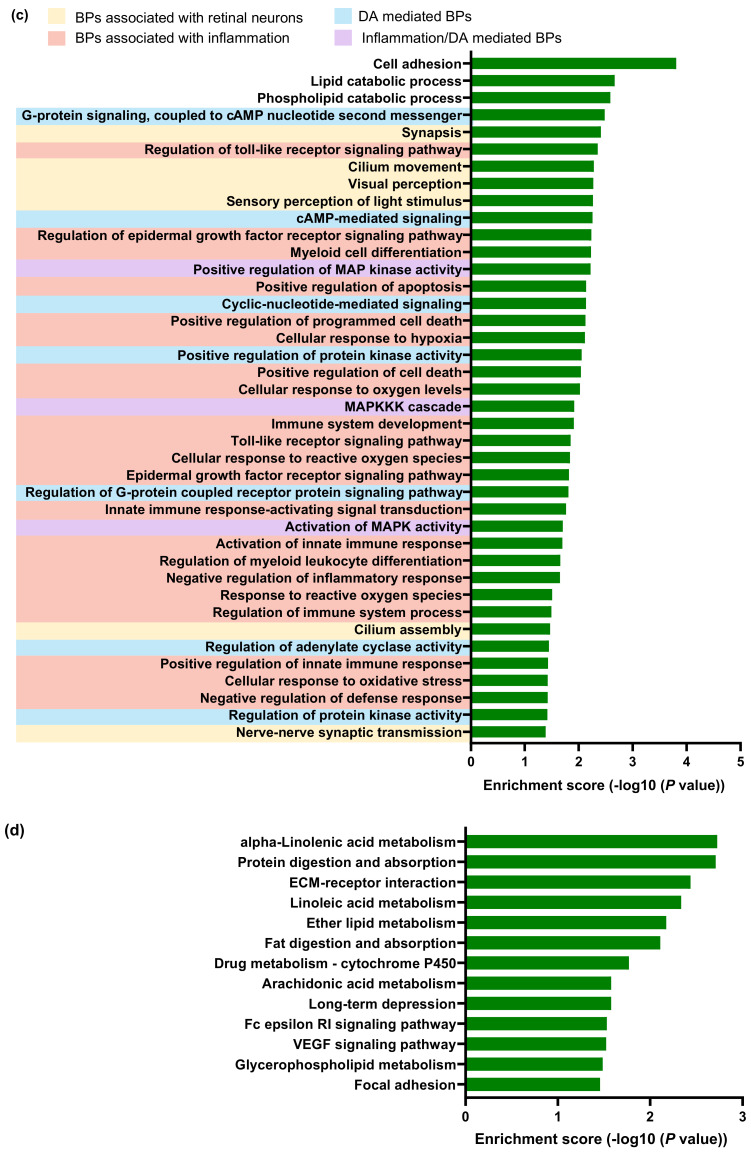
Gene functional annotation in *Gnat1^rd17^* mice by GO and KEGG. (**a**–**c**) GO enrichment and (**d**) KEGG pathway analysis of protein-coding DEGs in the neuroretina of *Gnat1^rd17^* mice vs. WT mice. The statistically significant GO terms (*p* 0.05) for (**a**) molecular function (MF), (**b**) cellular component (CC), and (**c**) biological process (BP) (40 selected GO terms). Individual GO terms associated with DA functions, photoreceptors/retinal neurons, and inflammation are color-coded on the left side of the ordinate. The ordinate represents the GO term or KEGG pathway name, and the abscissa represents the enrichment score calculated as the negative base-10 logarithm of the *p*-value (−log10 (*p*-value)). The smaller the *p*-value for the difference, the larger the −log10 (*p*-value).

GO terms and KEGG pathways were analyzed to classify the functions of the protein-coding DEGs. The GO analysis identified 20 significantly enriched terms for the molecular function (Figure 4a), 40 for the cellular component (Figure 4b), and 216 for the biological process (Figure 4c and Table S2). The KEGG analysis showed 13 significantly enriched pathways in *Gnat1^rd17^* mice (Figure 4d). The most enriched molecular function GO terms included “DA biding” (GO:0035240), “phospholipase A2 activity” (GO:0004623), “phospholipase activity” (GO:0004620), and “G-protein-coupled amine receptor activity” (GO:0008227) (Figure 4a; highlighted in blue), all of which are involved in DA-mediated cellular signaling [47,48,49]. Regarding the cellular component GO terms, several terms were linked to photoreceptors and/or retinal neurons (Figure 4b; highlighted in yellow), such as “photoreceptor inner segment” (GO:0001917), “photoreceptor outer segment” (GO:0001750), “nonmotile primary cilium” (GO:0031513), “primary cilium” (GO:0072372), “cilium” (GO:0005929), “neuron spine” (GO:0044309), and “dendritic spine” (GO:0043197). Consistent with these findings, several of the enriched biological process GO terms were also associated with functions of photoreceptors and/or retinal neurons (Figure 4c; highlighted in yellow), e.g., “sensory perception of light stimulus” (GO:0050953), “visual perception” (GO:0007601), “cilium movement” (GO:0003341), “cilium assembly” (GO:0042384), “nerve–nerve synaptic transmission” (GO:0007270), and “synapsis” (GO:0007129) and with DA signaling (Gnegy, 2012), including “cAMP-mediated signaling” (GO:0019933), “regulation of G-protein-coupled receptor protein signaling pathway” (GO:0008277), “regulation of adenylate cyclase activity” (GO:0045761), and “regulation of protein kinase activity” (GO:0045859) (Figure 4c; highlighted in blue). Interestingly, the results of the biological process GO terms showed that DEGs were also enriched in inflammatory pathways, such as “regulation of toll-like receptor signaling pathway” (GO:00341210), “regulation of epidermal growth factor receptor signaling pathway” (GO:0042058), “negative regulation of inflammatory response” (GO:0050728), “positive regulation of innate immune response” (GO:0045089), “innate immune response-activating signal transduction” (GO:0002758), “myeloid cell differentiation” (GO:0030099), “cellular response to oxidative stress” (GO:0034599), “positive regulation of apoptosis” (GO:0043065) and other related pathways (Figure 4c; highlighted in pink). Other enriched biological process terms in *Gnat1^rd17^* mice were associated with mitogen-activated protein kinase (MAPK; Figure 4c; highlighted in violet), a key regulator of inflammation including EAU [50,51,52], which can be regulated not only by a variety of pro-inflammatory factors [50] but also by DA [53,54,55,56,57]. In addition, “lipid catabolic process” (GO:0016042) and “phospholipid catabolic process” (GO:0009395) were found to be enriched in *Gnat1^rd17^* mice (Figure 4c). These results correspond with those of KEGG analysis that also highlighted alterations of lipid/phospholipid pathways (Figure 4d) such as the “alpha-linolenic acid metabolism” (mmu00592), “linoleic acid metabolism” (mmu00591), “ether lipid metabolism” (mmu00565), “fat digestion and absorption” (mmu04975), “arachidonic acid metabolism” (mmu00590), and “glycerophospholipid metabolism” (mmu00564). It is well known that these KEGG pathways are interconnected and linked to inflammation. 

Notably, arachidonic acid is synthesized from linoleic acid and is found in cellular membranes esterified to glycerophospholipids, from which it can be liberated by cytosolic phospholipase A2 and give rise to potent pro-inflammatory mediators including prostaglandins, leukotrienes, and thromboxanes [58,59]. Moreover, “Fc epsilon RI (high-affinity IgE receptor) signaling” (mmu04664) and “vascular endothelial growth factor (VEGF) signaling” (mmu04664), both known to be associated with the release of arachidonic acid pro-inflammatory metabolites [60,61], were other enriched KEGG pathways found in *Gnat1^rd17^* mice. Given that the retina is a rich source of DA and that several GO terms related to DA signaling pathways, including those involved in regulation of inflammatory processes, were enriched in the neuroretina of *Gnat1^rd17^* mice, we further addressed the role of DA in EAU exacerbation.

### 2.4. Gnat1^rd17^ Mice Show Low Retinal DA Levels and DRD4 Expression, Independent of EAU

**Figure 5:**
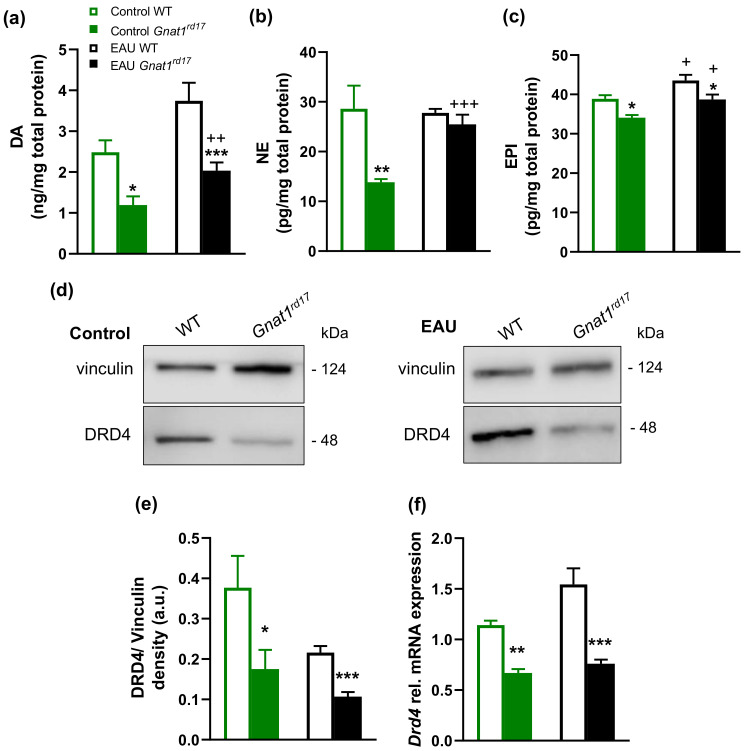
Alterations of the retinal catecholaminergic system in *Gnat1^rd17^* mice. (**a**) DA, (**b**) NE, and (**c**) EPI levels in the neuroretina of control (green bars) and EAU (black bars) mice (day 14 after immunization), both WT (open bars) and *Gnat1^rd17^* (filled bars). The data are presented as the mean ± SEM of 10 neuroretinas from 5 mice per group and were analyzed by one-way ANOVA followed by the Tukey–Kramer multiple comparison test. * *p* 0.05, ** *p* 0.01, and *** *p* 0.001: significant differences between WT and *Gnat1^rd17^* mice; ^+^ *p* 0.05, ^++^ *p* 0.01, and ^+++^ *p* 0.001: significant differences between control and EAU groups within WT and *Gnat1^rd17^* mice. The experiment was repeated twice with similar results. (**d**) Representative Western blot images and (**e**) densitometric analysis of DRD4 relative protein expression. Vinculin was used as a protein-loading control. The data represent the mean ± SEM of 5 to 6 neuroretinas from 5 to 6 control (green bars) and EAU (black bars) mice on day 14 after immunization, both WT (open bars) and *Gnat1^rd17^* (filled bars). The data shown are representative of three independent experiments. Differences between WT and *Gnat1^rd17^* groups: * *p* 0.05 and *** *p* 0.001 (unpaired *t*-test). (**f**) qRT-PCR results of mRNA expression for DRD4 in the neuroretinas of control (green bars) and EAU (black bars) mice on day 14 after immunization, both WT (open bars) and *Gnat1^rd17^* (filled bars). The data are presented as the mean ± SEM (unpaired *t*-test; n = 10 neuroretinas from 5 mice per each group; ** *p* 0.01 and *** *p* 0.001: differences between WT and *Gnat1^rd17^* groups).

**Figure 6:**
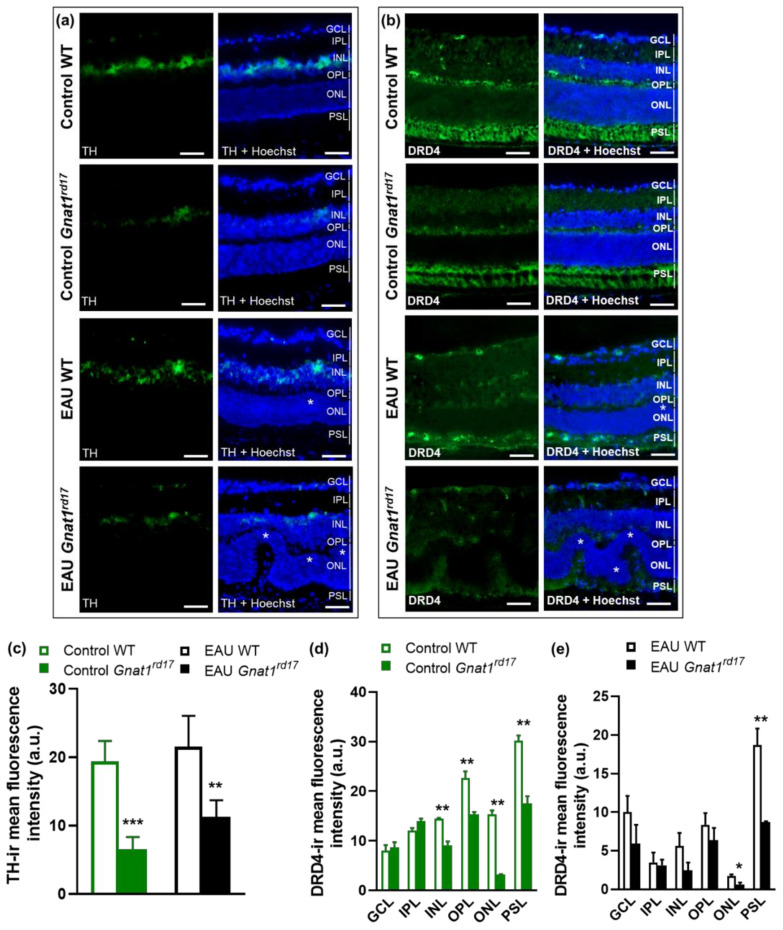
*Gnat1^rd17^* mice exhibit decreased TH and DRD4 protein expressions in the retina. Representative immunofluorescence staining (green) of (**a**) TH and (**b**) DRD4 protein expression and quantitative analysis of mean fluorescence intensities of (**c**) TH in the INL and (**d**,**e**) DRD4 expressions within all retinal layers in WT and *Gnat1^rd17^* mice, control and with EAU on day 14 after immunization, respectively. Retinal detachments that show retinal folds are indicated by white asterisks. Shown is one of two experiments with similar results. The data are presented as the mean ± SEM of 6 retinas from 6 mice per group. Differences between WT and *Gnat1^rd17^* groups: * *p* 0.05, ** *p* 0.01, and *** *p* 0.001 (unpaired *t*-test). Blue, Hoechst staining of nuclei. GCL, ganglion cell layer; IPL, inner plexiform layer; INL, inner nuclear layer; OPL, outer plexiform layer; ONL, outer nuclear layer; PSL, photoreceptor segment layer. The scale bar shows 50 μm.

In order to understand the possible involvement of DA in regulating EAU severity, we studied the extent of the alteration of DA production in *Gnat1^rd17^* mice. We observed significantly reduced DA concentrations in the neuroretinas of both healthy and EAU *Gnat1^rd17^* mice on day 14 after immunization (Figure 5a), which was accompanied by a decreased expression of tyrosine hydroxylase (TH), an essential enzyme for DA synthesis [49], in the retina (Figure 6a,c). Independently of EAU, the reduction in TH-immunoreactivity (TH-ir) was observed in the INL, where catecholaminergic amacrine cells (CA ACs) are normally found [62] (Figure 6a,c). The loss of amacrine cells leads to INL thinning. However, in *Gnat1^rd17^* mice, the thickness of the INL in the central and peripheral parts of the retina was similar to that of the control WT retina (Figure S5), suggesting that the reduced TH-ir and DA levels were not a result of CA AC degeneration. Because DA serves as a precursor to norepinephrine (NE) and epinephrine (EPI) [49], we next assessed whether a decline in DA levels is associated with decreased retinal levels of these catecholamines in *Gnat1^rd17^* mice. Indeed, healthy *Gnat1^rd17^* mice showed reduced retinal NE and EPI levels compared to healthy WT controls (Figure 5b,c). However, the retinal levels of all three catecholamines measured were significantly increased during EAU in *Gnat1^rd17^* mice compared to their healthy *Gnat1^rd17^* controls (Figure 5a–c). A slight increase in retinal EPI levels was also observed in EAU WT mice versus healthy WT controls (Figure 5c). Despite these changes associated with EAU, only retinal NE levels were similar in *Gnat1^rd17^* mice and WT mice during EAU (Figure 5b), and the *Gnat1^rd17^* mice with EAU still exhibited reduced retinal DA and EPI levels compared to EAU WT mice (Figure 5a,c). We also analyzed catecholamine levels in serum because they are released into systemic circulation in response to stress. We found no differences between *Gnat1^rd17^* and WT groups on any catecholamine measured (Figure S3a–c), implying no changes in stress response in any experimental groups that might affect the development of EAU.

To further characterize alterations in the retinal DA system in *Gnat1^rd17^* mice, we next focused on DA receptor subtype D4 (DRD4), which was found among the top 10 downregulated DEGs (Figure 3b, Table S1) in the neuroretina of *Gnat1^rd17^* mice. The qRT-PCR and Western blot analysis confirmed reduced DRD4 mRNA and protein expression in the neuroretina of *Gnat1^rd17^* mice, independently of EAU (Figure 5d–f and Figure S4b,c). Using fluorescence immunochemistry, we observed reduced DRD4-ir in the retinal ONL and photoreceptor segment layer (PSL) in EAU *Gnat1^rd17^*mice compared to EAU WT mice, whereas in healthy *Gnat1^rd17^* mice compared to WT mice, we found decreased DRD4-ir also in the INL and outer plexiform layer (OPL) (Figure 6b–e). A more prominent reduction of DRD4-ir in the retinal ONL during EAU, particularly in *Gnat1^rd17^* mice (Figure 6b–e), may be explained, at least in part, by the loss of photoreceptors, the main cells expressing DRD4 in the retina [63,64]. Indeed, histopathologically, retinal sections revealed more evident retinal folds and damage to the ONL in EAU *Gnat1^rd17^* mice than in EAU WT mice (Figure 6a,b; see asterisks).

The RNA-seq and qRT-PCR analysis also revealed downregulated expression of *Adrb2*, the gene-encoding beta 2-adrenergic receptor (β_2_-AR) protein, in the neuroretina of *Gnat1^rd17^* mice compared to WT mice (Table S1, Figure S3f). It is known that β_2_-AR signaling can be activated by DA [65] and plays an important role in the regulation of immune response in various autoimmune diseases [18,66]. Thus, we investigated whether changes in retinal β_2_-AR gene expression correlate with the protein expression. However, we observed that protein expression of β_2_-AR in the neuroretina did not differ between *Gnat1^rd17^* mice and WT mice under the condition with or without EAU (Figures S3d,e and S4d,e), which does not suggest modulation of β_2_-AR signaling in *Gnat1^rd17^* mice.

These results together indicate that rod dysfunction in both healthy and EAU *Gnat1^rd17^* mice results in loss of DA and suppressed DRD4-mediated activity, which may have implications for increased susceptibility to the development of severe EAU.

### 2.5. L-DOPA Treatment Attenuates EAU Severity in Gnat1^rd17^ Mice

**Figure 7:**
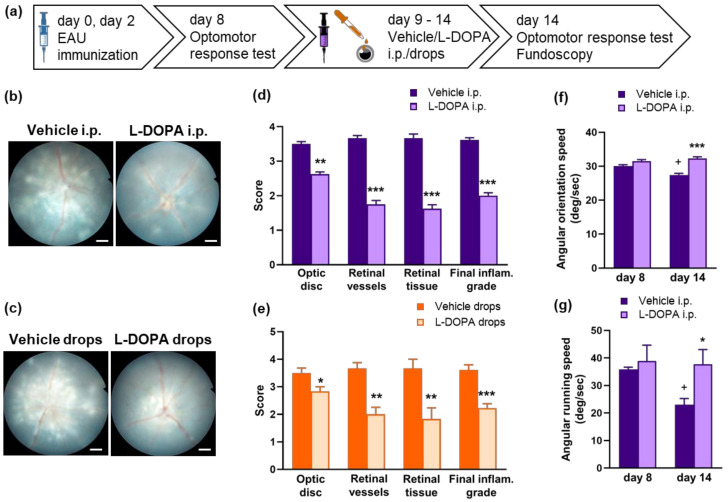
Systemic and topical administration of L-DOPA suppresses the development of severe EAU in *Gnat1^rd17^* mice. (**a**) Schematic of the experimental design. (**b**,**c**) Representative fundus images of EAU *Gnat1^rd17^* mice treated with vehicle or L-DOPA given i.p. or as eye drops. Fundus images were taken on day 14 after immunization. Scale bar: 200 μm. (**d**,**e**) EAU clinical score assessed on day 14 after immunization based on retinal inflammation observed in fundus images in vehicle- and L-DOPA-treated EAU *Gnat1^rd17^* mice. The data shown are the mean ± SEM of 6–8 eyes from 6–8 mice per group and are representative of two experiments. * *p* 0.05, ** *p* 0.01, and *** *p* 0.001: significant differences by unpaired *t*-test for comparisons between groups with i.p. injection of vehicle, and L-DOPA and paired *t*-test for comparisons between groups of vehicle-treated contralateral eyes and L-DOPA-treated ipsilateral eyes in the same animals. Optomotor responses: (**f**) angular orientation speed and (**g**) angular running speed in EAU *Gnat1^rd17^* mice that were treated with vehicle or L-DOPA i.p. injection, measured in the optomotor drum on days 8 and 14 after immunization. Data shown are the mean ± SEM of 4 mice in each group; one representative out of two experiments is shown. * *p* 0.05 and *** *p* 0.001 by unpaired *t*-test for comparison between vehicle- and L-DOPA-treated EAU *Gnat1^rd17^* groups; ^+^ *p* 0.05 by paired *t*-test for comparison between days 8 and 14 within the same group.

**Figure 8:**
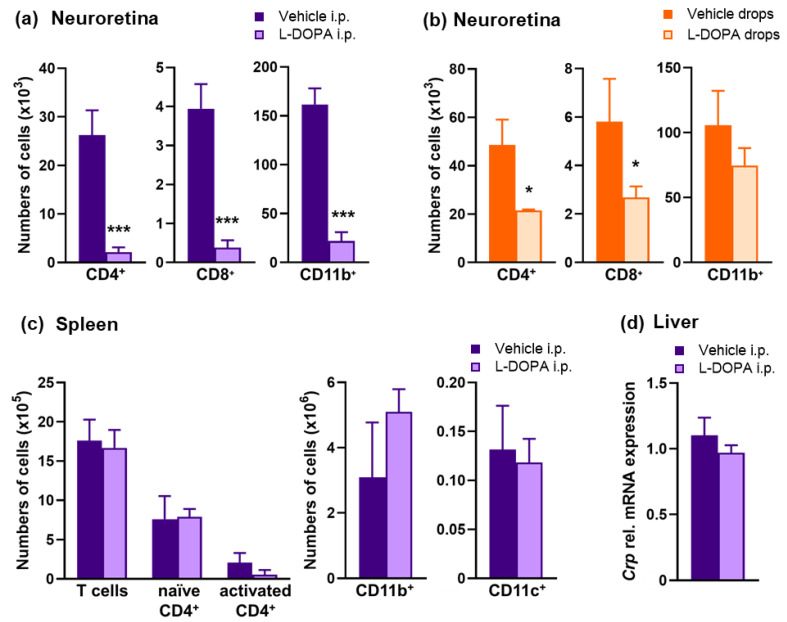
L-DOPA treatment in EAU *Gnat1^rd17^* mice decreases retinal infiltration of immune cells. Absolute numbers of neuroretina-infiltrating CD4^+^, CD8^+^, and CD11b^+^ cells in EAU *Gnat1^rd17^* mice treated with vehicle or L-DOPA given (**a**) i.p. or as (**b**) eye drops (day 14 after immunization) were measured by flow cytometry. The data shown are the mean ± SEM of 6–8 neuroretinas from 6–8 mice per group and are representative of two experiments. * *p* 0.05 and *** *p* 0.001 by unpaired *t*-test for comparisons between groups receiving i.p. injections of vehicle and L-DOPA, respectively, and paired *t*-test for comparisons between groups of vehicle-treated contralateral eyes and L-DOPA-treated ipsilateral eyes in the same animals. (**c**) Absolute numbers of CD3^+^ T cells, naïve CD4^+^ T cells (CD4^+^CD44^low^), activated CD4^+^ T cells (CD4^+^CD44^high^), CD11b^+^ (CD11b^high^CD11c^low^, macrophages, and neutrophils), and CD11c^+^ (CD11c^high^MHC-II^high^, dendritic cells) in spleens of EAU *Gnat1^rd17^* mice treated with i.p. injections of vehicle or L-DOPA. Flow cytometry was used to analyze the data on day 14 after immunization, and the results are shown as the means ± SEM of 4 mice in each group (unpair *t*-test). Shown is one representative experiment of two independently performed experiments. (**d**) qRT-PCR analysis of *Crp* mRNA levels in the liver of EAU *Gnat1^rd17^* mice treated with i.p. injections of vehicle or L-DOPA. Data were collected on day 14 following immunization and are presented as the mean ± SEM of 4 mice in each group (unpair *t*-test).

In our subsequent experiments, we aimed to test whether supplementing retinal DA with L-DOPA could prevent the development of severe EAU in *Gnat1^rd17^* mice. We observed that daily treatment with L-DOPA intraperitoneal (i.p.) injection from day 9 after immunization (preclinical phase of EAU) [21] significantly reduced the EAU clinical score and prevented the deterioration of visual acuity measured by optomotor response in *Gnat1^rd17^* mice on day 14 after immunization (Figure 7b,d,f,g). At the same time, i.p. L-DOPA treatment markedly decreased absolute numbers of infiltrated CD4^+^ T cells, CD8^+^ T cells, and CD11b^+^ cells in the neuroretina (Figure 8a). To gain further understanding of whether the systemic effects of L-DOPA might contribute to reduced immune-cell infiltrates, we examined peripheral immune cells collected from the spleens of EAU *Gnat1^rd17^* mice treated with L-DOPA. We quantified the number of splenic CD3^+^ T cells, activated/memory and naïve CD4^+^ T cells, CD11b^+^ cells, and CD11c^+^ cells. We observed similar numbers of these splenic cell populations in L-DOPA-treated and vehicle-treated EAU *Gnat1^rd17^* mice (Figure 8c). Similarly, no differences were found for *Crp* mRNA expression in the liver of L-DOPA-treated and vehicle-treated EAU *Gnat1^rd17^* mice (Figure 8d). It therefore seems unlikely that i.p.-administered L-DOPA significantly influenced systemic inflammation in EAU *Gnat1^rd17^* mice.

In addition, to confirm the direct effect of L-DOPA on the retina, L-DOPA was also applied topically in the form of eye drops. We observed that the treatment with L-DOPA eye drops administered twice daily from day 9 after immunization had similar efficacy as the treatment with i.p. L-DOPA, especially in suppressing the clinical score of the disease (Figure 7c,e) and the infiltration of CD4^+^ and CD8^+^ T cells in the neuroretina (Figure 8b). However, compared with L-DOPA i.p. treatment, L-DOPA eye drops showed only a mild effect on the retinal recruitment of CD11b^+^ cells in EAU *Gnat1^rd17^* mice (Figure 8b). 

Taken together, these data show that either systemic or local application of L-DOPA effectively decreases the pathological degree of EAU and retinal inflammation in *Gnat1^rd17^* mice.

### 2.6. L-DOPA Treatment Rerestored Reduced Retinal DA Levels in EAU Gnat1^rd17^ Mice

**Figure 9:**
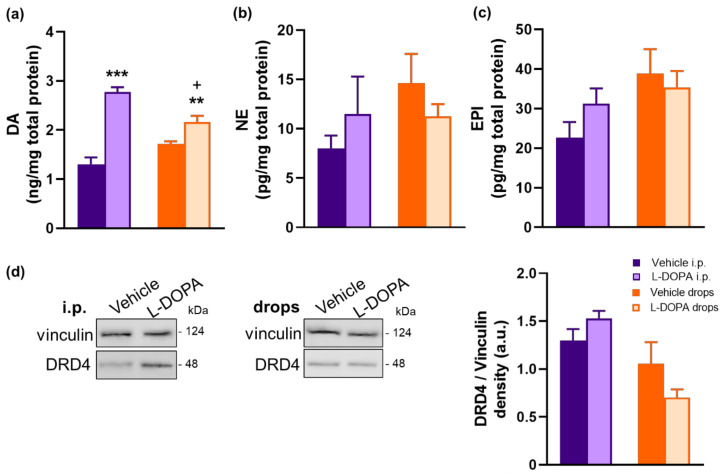
L-DOPA treatment increases retinal DA levels in *Gnat1^rd17^* mice. (**a**) DA, (**b**) NE, and (**c**) EPI levels in the neuroretina measured by ELISA in EAU *Gnat1^rd17^* mice treated with vehicle (light purple and light orange bars) or L-DOPA (dark purple and dark orange bars) given i.p. (purple bars) or topically (orange bars) in the form of eye drops. The data are shown as the mean ± SEM of 8 neuroretinas from 4–6 animals per group. Significant differences between groups: *** *p* 0.001 by unpaired *t*-test for comparisons of vehicle and L-DOPA i.p. groups, ** *p* 0.01 by paired *t*-test for comparisons of vehicle-treated contralateral eyes and L-DOPA-treated ipsilateral eyes in the same animals, and ^+^ *p* 0.05 by unpaired *t*-test for comparisons of L-DOPA i.p. vs. L-DOPA drops. (**d**) Left: representative Western blots for DRD4 expression in neuroretinas of EAU *Gnat1^rd17^* mice treated with vehicle or L-DOPA given i.p. or in the form of eye drops. Right: densitometric analysis of relative protein expressions of DRD4. Vinculin was used as a protein-loading control. Results are presented as the mean ± SEM. No significant differences were found after treatment with L-DOPA.

In our next set of experiments, we investigated the functional roles of L-DOPA in replacing DA, NE, and EPI in the retina and in regulating retinal DRD4 expression. We observed that both L-DOPA i.p. injection and L-DOPA eye drops increased retinal DA levels in EAU *Gnat1^rd17^* mice (Figure 9a). However, when compared to L-DOPA eye drops, the effect of L-DOPA i.p. injection on retinal DA levels was stronger (Figure 9a), which could have been due to the lower bioavailability of L-DOPA from the eye drops and could explain the better efficacy of L-DOPA administered systemically versus topically in reducing immune-cell infiltration in the neuroretina (Figure 8a,b). On the other hand, retinal NE and EPI levels remained unaffected following the L-DOPA challenge (Figure 9b,c), which may reflect the fact that retinal expression of the DA synthetic enzyme DOPA decarboxylase is several-fold higher than expression of DA-β-hydroxylase and phenylethanolamine N-methyltransferase, the enzymes required for NE and EPI synthesis, in the CA ACs in the mouse retina [62].

Compared with vehicle treatment, neither i.p. nor topical L-DOPA treatment caused moderate but not significant changes in retinal DRD4 protein expression in EAU *Gnat1^rd17^* mice (Figure 9d and Figure S4f,g). These data suggest that L-DOPA-induced increased retinal DA content is not accompanied by alterations in the retinal DRD4 expression to elicit protective effects against the development of severe EAU.

### 2.7. The Anti-Inflammatory Effects of L-DOPA/DA in EAU Gnat1^rd17^ Mice Are Mediated by the Suppression of NF-κB (p65) and STAT3 Activity in Retinal Endothelial Cells

**Figure 10:**
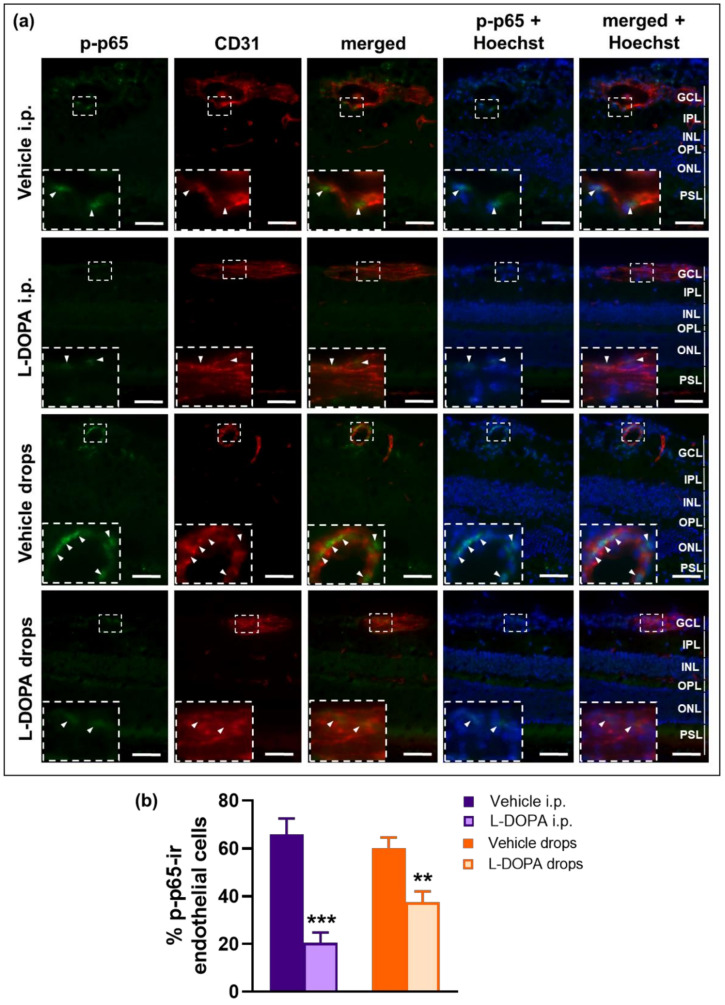

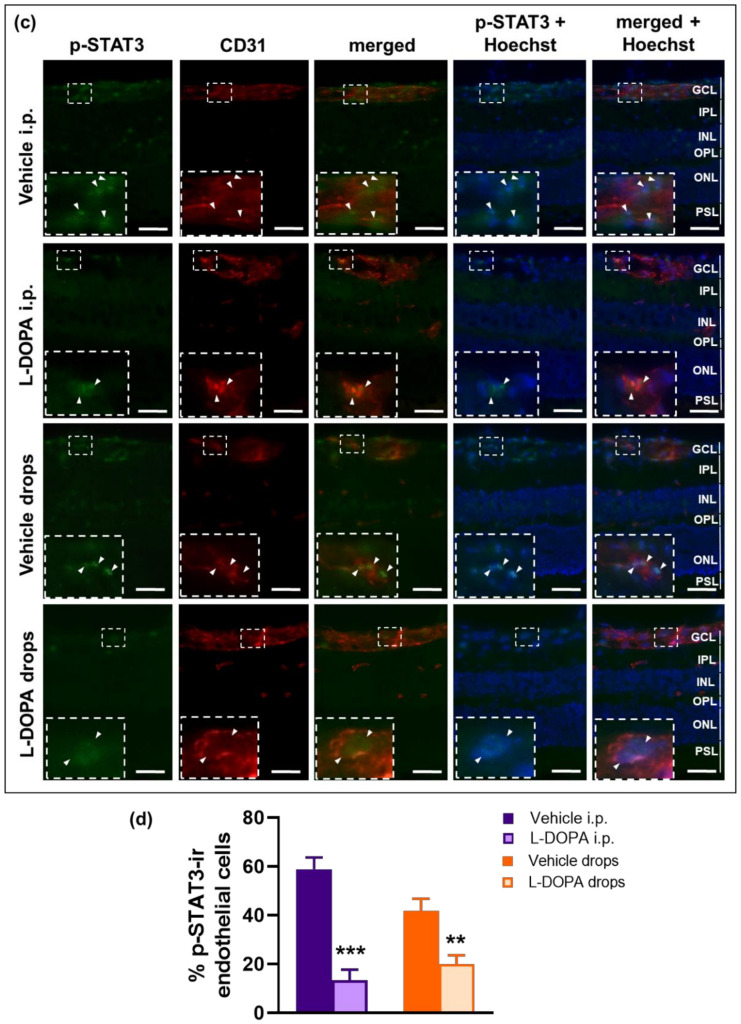
EAU *Gnat1^rd17^* mice show decreased p-p65 and p-STAT3 expression in retinal endothelial cells after L-DOPA treatment. Representative transverse retinal sections of EAU *Gnat1^rd17^* mice (day 14 after immunization) treated with vehicle or L-DOPA given i.p. or topically as eye drops were co-immunolabeled for (**a**) p-p65 (green) or (**c**) p-STAT3 (green) and CD31 (red) to stain endothelial cells. Hoechst staining (blue) indicates nuclei. The scale bar is 50 μm. Insets show higher magnification of p-p65-immunoreative (p-p65-ir) or p-STAT3-immunoreactive (p-STAT3-ir) endothelial cells (marked with arrowheads). Bar graphs show the mean percentage of (**b**) p-p65-ir or (**d**) p-STAT3-ir endothelial cells per total number of endothelial cells within one section. The data are expressed as the mean ± SEM of 6 retinas from 6 animals per group. ** *p* 0.01 by paired *t*-test for comparisons between groups of vehicle-treated contralateral eyes and L-DOPA-treated ipsilateral eyes in the same animals, and *** *p* 0.001 by unpaired *t*-test for comparisons between groups with i.p. injection of vehicle and L-DOPA. GCL, ganglion cell layer; IPL, inner plexiform layer; INL, inner nuclear layer; OPL, outer plexiform layer; ONL, outer nuclear layer; PSL, photoreceptor segment layer.

The pro-inflammatory cytokines IL-6 and IL-17A are known to play a crucial role in the pathogenesis of EAU [67]. These cytokines synergistically activate NF-κB and STAT3, resulting in enhanced expression of inflammatory chemokines in endothelial cells to recruit immune cells across the BBB or BRB into the central nervous system or retina, respectively [22,23,24,25,26,27]. Based on our previous study [21], we hypothesized that the protective effect of L-DOPA on suppressing the recruitment of immune cells into the retina could be mediated by the inhibition of NF-κB and STAT3 activity in the BRB endothelium. Phosphorylation of NF-κB subunit p65 at Ser276 is a marker of NF-κB activation and an essential prerequisite for transcriptional activity of p65 and expression of its downstream target genes [68,69]. A marker of STAT3 activation is the phosphorylation of Tyr705, which is responsible for STAT3 dimerization and translocation to the nucleus [70]. Therefore, we determined the effect of L-DOPA treatment on phosphorylation of NF-κB p65 at Ser276 (p-p65) and STAT3 phosphorylation at Tyr705 (p-STAT3) in retinal endothelial cells using double immunofluorescence staining on retinal sections. We detected that the percentage of p-p65-ir endothelial cells (Figure 10a,b) and p-STAT3-ir endothelial cells (Figure 10c,d) were markedly decreased in the retinal microvasculature after both systemic and local L-DOPA treatments in EAU *Gnat1^rd17^* mice. 

**Figure 11:**
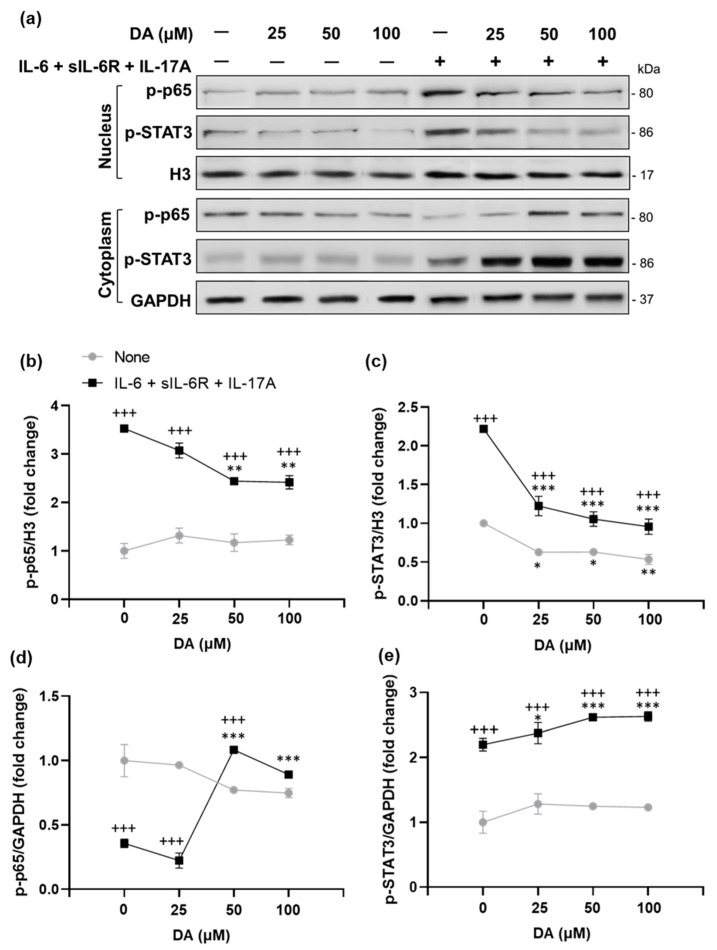

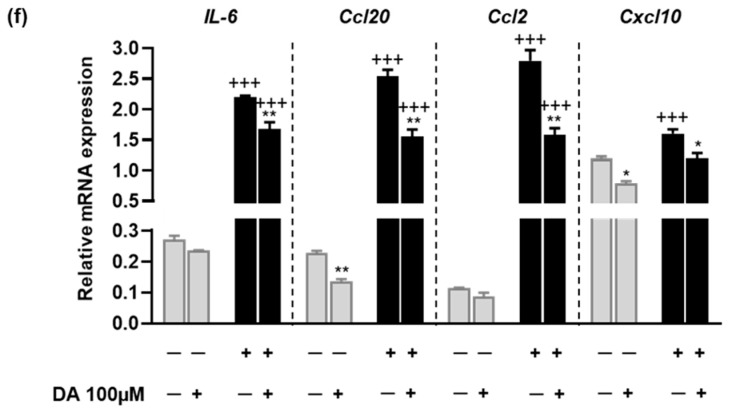
DA suppresses the inflammatory response by inhibiting nuclear localization of p-p65 (NF-κB) and p-STAT3 and expression of their target genes in rat retinal capillary endothelial cells. (**a**) Representative Western blots and densitometric analysis of p-p65 and p-STAT3 in the (**b**,**c**) nuclear and (**d**,**e**) cytoplasmic fractions of rat retinal capillary endothelial cells treated with or without DA (25, 50, and 100 M) under the basal condition (none; grey line) or stimulation with IL-6, sIL-6R, and IL-17A. Results are presented as a fold change to control (basal condition without DA pretreatment) after normalizing with histone H3 in nuclear fractions and GAPHD in cytoplasmic fractions. (**f**) The mRNA expression of *IL-6*, *Ccl20*, *Ccl2*, and *Cxcl10* measured by qRT-PCR. GAPDH was used as an endogenous control. The data are the means ± SEM of three biological replicates. * *p* 0.05, ** *p* 0.01, and *** *p* 0.001 by unpaired *t*-test analysis for comparisons between the DA treated group and the corresponding DA untreated group, and ^+++^ *p* 0.001 by unpaired *t*-test analysis for comparisons between the basal and cytokine-stimulated conditions with the corresponding concentration of DA.

In addition, we investigated the effect of DA on pro-inflammatory cytokine (IL-6, sIL-6R, and IL-17A)-induced p-p65 and p-STAT3 expression in rat retinal capillary endothelial cells. We detected that DA pretreatment in a dose-dependent manner significantly decreased nuclear p-p65 and p-STAT3 expressions (Figure 11a–c and Figure S4h,i,l,m), which was accompanied by increased expressions of these transcription factors in the cytoplasm Figure 11a,d,e and Figure S4j,k,n,o). Our analysis also revealed that DA decreased nuclear p-STAT3 expression even under a basal condition, without stimulation with cytokines (Figure 11a,c and Figure S4l ). Since NF-κB and STAT3 activate numerous pro-inflammatory genes that facilitate the recruitment of CD4^+^ T cells in the retina to initiate the inflammatory process, we evaluated mRNA expression of *IL-6* and T-cell-attracting chemokines such as *Ccl20*, *Ccl2*, and *Cxcl10* under the basal condition and under the condition of stimulation with IL-6/sIL-6R/IL-17A in DA pretreated retinal endothelial cells. As shown in Figure 11f, DA reduced mRNA expression of *IL-6* and all measured chemokines in cells stimulated with IL-6/sIL-6R/IL-17A. Under the basal conditions, there was a trend towards the inhibition of *IL-6* and chemokine expressions after DA pretreatment. However, the significantly suppressed expressions were detected only for *Ccl20* and *Cxcl10* genes (Figure 11f).

Overall, these observations demonstrate that L-DOPA/DA acts to ameliorate EAU by decreasing NF-κB (p65) and STAT3 activation and expression of their pro-inflammatory target genes in the endothelial cells of retinal vessels, protecting the BRB from inflammatory damage.

## 3. Discussion

We identified “DA binding” as the most significantly deregulated molecular function GO term together with decreased retinal TH expression and DA levels in 10-week-old *Gnat1^rd17^* mice, suggesting that dysfunction of the retinal dopaminergic system is one of the early changes in *Gnat1^rd17^* mice that occur long before any abnormalities are detected in the retinal structure [30,33]. Although our study is the first to explore the retinal dopaminergic system in *Gnat1^rd17^* mice, previous studies also investigated DA changes that are associated with Gnat1 deficiency. *Gnat1^−/−^* mice, which show early mild photoreceptor cell loss of about 10% [33,71,72], were reported to exhibit a significant decrease in the number of TH-expressing cells, reduced DA levels, and a low concentration of the DA metabolite 3,4-dihydroxyphenylacetate (DOPAC) in the retina [73,74,75]. According to recent findings, the decreased production of DA in the retina under conditions of Gnat1 deficiency can be explained by the fact that rod phototransduction, which is Gnat1-dependent, stimulates dopaminergic amacrine cells (CA type 1 ACs; CAI ACs) through bipolar cell synapses [74,76]. Therefore, the absence of normal Gnat1 in *Gnat1^rd17^* mice, just as in *Gnat1^−/−^* mice, can explain the decrease in retinal DA. The other issue to consider in the reduction of retinal DA levels is rod photoreceptor degeneration in the EAU model induced by active immunization with IRBP. The IRBP constitutes most of the extracellular matrix present between the photoreceptors and the retinal pigment epithelium [77]. Thus, in this EAU model, the infiltration of pathogenic CD4+ T cells that mediate an autoimmune response against IRBP, combined with the accumulation of subretinal fluid caused by BRB breakdown, results in the destruction of photoreceptors [28,29,77,78,79]. However, although the EAU-induced disruption of the retinal ONL structure, reflecting photoreceptor loss, was extensive in *Gnat1^rd17^* mice, the retinal DA levels were higher in EAU *Gnat1^rd17^* mice compared to age-matched *Gnat1^rd17^* mice without EAU and signs of photoreceptor degeneration. These findings indicate that the involvement of a rod photoreceptor-independent mechanism may promote a modest increase in retinal DA levels in *Gnat1^rd17^* mice during EAU.

In this study, we showed that *Gnat1^rd17^* mice with dysfunctional rod phototransduction develop exacerbated EAU due to a lack of retinal DA production, which contributes to a pro-inflammatory phenotype of retinal endothelial cells via NF-κB and STAT3 activation. Our study also demonstrated the protective effect of L-DOPA as a DA replenishing substance in the retina to prevent the exacerbation of EAU in *Gnat1^rd17^* mice by both systemic and eye-drop application early in the course of EAU development. These findings, together with our previous report showing that light-mediated ocular NE and EPI release prevent excessive expression of chemokines in the retina via desensitization of the alpha 1-AR (α_1_-AR) receptor, leading to the suppression of immune-cell infiltration and amelioration of EAU [21], highlight the importance of neural regulation of the dynamic nature of the BRB in the modulation of EAU pathogenesis. 

Our results suggest that rod phototransduction also plays a role in releasing retinal NE and EPI levels. Considering DA as a precursor of NE and EPI [49], we reasoned that the depletion of NE and EPI levels in the retina of *Gnat1^rd17^* mice was attributed to low DA levels. Retinal NE and EPI are mainly synthetized by CA ACs type 2 (CAII ACs) from DA released in the retina by CAI ACs [62], since the retina, including the intraocular portion of retinal vessels, does not receive sympathetic input [80]. Nevertheless, under EAU conditions, it appears that retinal NE and EPI levels do not solely depend on the DA content in the retina, as retinal NE levels in *Gnat1^rd17^* mice returned to control values and only a slight decrease in retinal EPI levels was detected in *Gnat1^rd17^* mice compared to controls during EAU. This suggests either an inflammatory regulation of retinal NE/EPI synthesis by CAII ACs or an extraretinal origin of NE and EPI from peripheral circulation due to the BRB disruption or from infiltrated immune cells, which are also known to produce catecholamines [81]. Regarding NE and EPI and their immunomodulatory properties, generally, the bulk of evidence points to anti-inflammatory effects through β-ARs and pro-inflammatory effects through α-ARs in autoimmune diseases [82,83]. Our previous findings on the amelioration of EAU by a photopic light-induced increase in retinal NE and EPI levels along with downregulation/desensitization of α_1_-AR also support this concept [21]. However, our current study does not suggest alterations of retinal ARs in EAU *Gnat1^rd17^* mice. Even though the *Adrb2* gene encoding β_2_-AR was identified to be significantly downregulated in the neuroretina of *Gnat1^rd17^* mice, we did not detect a decrease in retinal β_2_-AR protein levels in healthy or EAU *Gnat1^rd17^* mice compared to their WT controls. Thus, our results rule out a role for the retinal noradrenergic system in regulating the pathogenesis of EAU in *Gnat1^rd17^* mice and underscore the importance of the retinal dopaminergic system in this process based on the reduced TH expression, DA levels, and DRD4 gene and protein expression in the retina of healthy and EAU *Gnat1^rd17^* mice.

Suppressed retinal DA pathways with aberrant visual function consequences were also observed in animal models of hereditary retinal degeneration associated with photoreceptor apoptosis, such as the Royal College of Surgeons (RCS) rats and retinal degeneration slow (*rds*) mice prior to the onset of photoreceptor cell loss [84]. Regarding the direct effect of DA on rod photoreceptors, there is a controversy between in vitro and in vivo tests. In vitro results in retinas harvested from retinal dystrophic (*rd)* mice showed an increase in photoreceptor survival after treatment with DA antagonists or DA depletion, which was not reproduced in vivo by either a pharmacological or genetic approach [85,86]. On the other hand, enhancement of DA receptor DRD1 activity in a murine model of light-induced retinal degeneration protected Müller cells from injury, which is an important aspect for the protection of the BRB, as Müller cells interact with both neurons and endothelial cells [87]. In light of these previous studies, the *Gnat1^rd17^* mice, characterized by late-onset retinal degeneration after the age of 6 months, presented abnormalities in the retinal DA system that we observed at the age of 8–10 weeks, which may be a marker of a pre-degeneration stage and may have a significant impact on retinal homeostasis, including the BRB breakdown.

Given the role of DA signaling via DRD4 receptor in the retina, which is known to be expressed particularly in photoreceptors [63,64], the pathological mechanisms resulting from downregulation of the DA/DRD4 pathway in *Gnat1^rd17^* mice might include cone mispositioning in the developing retina [64], dysfunctional adaptation to changing environmental illumination, and disruption of the circadian rhythm of photoreceptor AC1 activity and cAMP production [88,89]. DRD4 belongs to the D2-like receptors, which are coupled to the Gα_i/o_ protein, inhibit adenylyl cyclase (AC), and reduce the intracellular concentration of cyclic adenosine monophosphate (AMP), which blocks PKA activity [90]. Consistent with the low retinal DRD4 expression, we also observed that GO terms associated with DRD4 signaling, such as “G-protein coupled amine receptor activity,” “cAMP-mediated signaling,” “cyclic nucleotide-mediated signaling,” and “regulation of adenylate cyclase activity,” were significantly deregulated in the neuroretina of *Gnat1^rd17^* mice. In addition, activation of DRD4 has also been reported to regulate MAPK signaling pathways, which play an important role in immune responses [53,54,55,56,57]. In this respect, our results showed significantly enriched biological processes for GO terms including “positive regulation of MAP kinase activity,” “MAPKKK cascade,” and “activation of MAPK activity” in the neuroretina of *Gnat1^rd17^* mice, which may provide another mechanism resulting from suppressed retinal DA/DRD4 signaling. However, there are conflicting reports regarding the DRD4 effects on MAPK pathways, showing either activation [53,54,55] or inhibition [56,57], which may depend on the cell type. Therefore, further studies are warranted to investigate precise interactions between DA/DRD4 and MAPK signaling in the retina.

Finally, our evaluation of the functional significance of retinal DA replacement using L-DOPA systemic or topical administration in the form of eye drops revealed a protective effect of DA against EAU exacerbation in *Gnat1^rd17^* mice. The beneficial properties of L-DOPA have also been demonstrated in previous experimental and clinical studies in other ocular diseases associated with retinal inflammation, including diabetic retinopathy [91,92,93] and age-related macular degeneration [94]. The importance of the dopaminergic system has also been shown in endotoxin-induced uveitis, in which a mixed DRD2 agonist/DRD1 antagonist was able to reduce immune-cell infiltrates in the eye [95]. In our study we did not assess which DA receptors mediated anti-inflammatory effects of L-DOPA. Nevertheless, we provide evidence that the downregulation of the retinal DRD4 expression *Gnat1^rd17^* mice remained unchanged following L-DOPA treatment, suggesting that the mechanism by which L-DOPA attenuates EAU does not rely on restored DRD4 expression in the *Gnat1^rd17^* retina. This finding also demonstrates that regulation of DRD4 expression in the retina of *Gnat1^rd17^* mice is more complex and does not depend only on the availability of DA as a receptor ligand.

At the molecular level, we found that the mechanism of action of L-DOPA involves suppression of NF-κΒ and STAT3 activity in retinal endothelial cells of EAU *Gnat1^rd17^* mice. These results were recapitulated in vitro using rat retinal capillary endothelial cells stimulated with pro-inflammatory cytokines after DA pretreatment. Specifically, DA pretreatment dose-dependently inhibited nuclear translocation of the NF-κΒ p-p65 subunit and p-STAT3, which was accompanied by decreased expression of NF-κΒ and STAT3 target genes encoding IL-6 and chemokines involved in T-cell recruitment. Since the recruitment of autoreactive CD4^+^ T cells into the retina is a critical hallmark of EAU pathogenesis [78,79], L-DOPA-/DA-mediated reduced activity of NF-κΒ and STAT3 in retinal vascular endothelial cells represents an important mechanism inhibiting BRB permeability to autoreactive CD4^+^ T cells that initiate the development of EAU. The DA-induced suppression of NF-κΒ and STAT3 activity is not specific only to endothelial cells, as it has also been demonstrated in other cells, including chondrocytes, glioma cells, and hepatocytes [96,97,98]. Moreover, the protective effects of DA on endothelial cells might also include other mechanisms that were not tested in this study, but were reported previously, such as inhibition of apoptosis, oxidative stress [99,100], VEGF receptor 2, focal adhesion kinase, and MAPK in endothelial cells [101], as well as suppression of microvascular hyperpermeability, angiogenesis, proliferation, and migration of endothelial cells [102].

In the present study, we used *Gnat1^rd17^* mice as a model of rod dysfunction associated with late-onset rod–cone dystrophy to elucidate a possible mechanism by which RP creates conditions predisposing to AU. Although we chose to investigate the role of the retinal neural network alterations in the modulation of the BRB endothelium in EAU, more mechanisms might be involved in EAU pathogenesis in *Gnat1^rd17^* mice. These mechanisms can be associated with enriched GO terms related to oxidative stress, innate immune response, apoptosis, lipid and arachidonic acid metabolism, synaptic functions, photoreceptor segments, and cilia that we identified in the neuroretina of *Gnat1^rd17^* mice. Regarding the latter enriched GO terms, the differential expression of *Ush1c* and *Myo7a* genes coding for Usher proteins in the neuroretina of *Gnat1^rd17^* mice is particularly interesting because of their location and function in the connecting cilium and synaptic region of photoreceptors and the association of their mutations with RP [10,103]. In addition, we identified *Cwc22* as the most upregulated gene in the neuroretina of *Gnat1^rd17^* mice. The biological importance of CWC22 in the retina is not completely understood, but it interacts with the splicing factor CWC27, involved in retinal dysfunction and degeneration [45,46]. Interestingly, the overexpression of *Cwc22* was also observed in sensory neurons of diabetic mice, where it was found to act as a mediator of diabetic polyneuropathy [104]. Therefore, the abovementioned deregulated GO terms and genes might be significant for susceptibility to retinal degeneration and inflammation in *Gnat1^rd17^* mice. The characterization of their specific roles and regulation in the retina of *Gnat1^rd17^* mice is beyond the scope of the present study. However, the identification of these GO terms and genes raises questions for further investigation of the model of late retinal degeneration in *Gnat1^rd17^* mice.

In summary, our findings show that rod dysfunction in *Gnat1^rd17^* mice increases susceptibility to severe EAU via downregulation of the retinal dopaminergic system, which, under physiological conditions, serves as an anti-inflammatory gateway reflex, protecting against the establishment of gateways across the BRB for immune-cell infiltration into the retina. DA supplementation by L-DOPA suppresses a pro-inflammatory phenotype in retinal endothelial cells by inhibiting NF-κΒ and STAT3 activity, thereby reducing the expression of chemokines that attract immune cells into the retina. Thus, L-DOPA treatment may represent a promising strategy to reduce the risk of severe AU in rod dysfunction or other diseases associated with retinal DA deficiency. 

## 4. Materials and Methods

### 4.1. Animals

The *Gnat1^rd17^* mouse (B6.Cg-*Gnat1^irdr^*/Boc, Stock No: 008811, retinal degeneration 17), homozygotes for a mutant *Gnat1^irdr^* (ICR-derived retinal dysfunction rod) allele that was placed on the background of strain C57BL/6J [30] were purchased from The Jackson Laboratory (Bar Harbor, ME, USA) and were brother-x-sister mated for one generation and their offspring were used in experiments. Wild-type (WT) C57BL/6J mice obtained from Charles River Laboratories (Sulzfeld, Germany) served as genetic background controls. At the time of the experiment, the mice were between the ages of 8 and 10 weeks. All mice were males except in the L-DOPA studies, where both male and female mice were used in approximately equal numbers. The mice were housed in the animal facility at the Department of Physiology, Third Faculty of Medicine, Charles University, under a 12 h/12 h light/dark cycle (lights on at 7:30 a.m. and off at 7:30 p.m.) in a temperature- (23 ± 1 °C) and humidity (50 ± 10%)-controlled environment and provided with nesting enrichment and free access to standard rodent diets and drinking water.

### 4.2. Cell Culture 

The immortalized rat retinal capillary endothelial cells SV40 (T9097) and the corresponding applied cell extracellular matrix (G422) and Prigrow IV medium (TM004) supplemented with 10% fetal bovine serum (FBS) were obtained from Applied Biological Materials (Richmond, BC, Canada). The cells were seeded into 24-well plates at 50,000 cells/well and pretreated with dopamine hydrochloride (25, 50, and 100 μM; Tocris Bioscience, Bristol, UK) for 2 h followed by a 24 h treatment with or without cytokine cocktail containing IL-6 (100 pg/μL; Thermo Fisher Scientific, Waltham, MA, USA), sIL-6R (100 pg/μL; Thermo Fisher Scientific, Waltham, MA, USA), and IL-17A (50 pg/μL; Thermo Fisher Scientific, Waltham, MA, USA). The cells were then processed for RNA isolation and qRT-PCR or cytoplasmic and nuclear protein extractions using Minute™ Cytoplasmic and Nuclear Extraction Kit for Cells (Invent Biotechnologies, Plymouth, MN, USA) for Western blotting.

### 4.3. EAU Induction 

EAU was induced in WT and *Gnat1^rd17^* mice by active immunization with emulsion containing 200 µg of human IRBP_1–20_ (interphotoreceptor retinoid-binding protein; amino acid sequence of peptide (1–20): GPTHLFQPSLVLDMAKVLLD; custom product by Sigma-Aldrich, Merck KGaA, Darmstadt, Germany) and complete Freund’s adjuvant (CFA) composed of 1 mg Mycobacterium tuberculosis strain H37Ra (Becton, Dickinson and Company, Franklin Lakes, NJ, USA) and incomplete Freund’s adjuvant (Sigma-Aldrich, Darmstadt, Germany). The emulsion of IRBP_1–20_ in CFA (100 μL) was injected subcutaneously into the base of the tail (day 0), and the mice were injected intravenously with 200 μL of 0.2 μg pertussis toxin from Bordetella pertussis (Sigma-Aldrich, Darmstadt, Germany) in 0.9% saline on days 0 and 2. 

### 4.4. L-DOPA Treatment

The effect of retinal DA replacement was investigated by treating *Gnat1^rd17^* mice with L-DOPA (3,4-dihydroxy-L-phenylalanine, D9628, Sigma-Aldrich, Darmstadt, Germany) using different administration routes: i.p. injection and topical application of eye drops. For i.p. administration, L-DOPA was dissolved in 0.9% saline just prior to injection and was given in a dose of 25 mg/kg body weight per day at 7:30 a.m. from day 9 to day 14 after immunization. A control group received equal amounts of vehicle solution (0.9% saline). To increase the bioavailability of topically applied ophthalmic drugs, L-DOPA was dissolved to 15 mM in 1× phosphate-buffered saline (PBS, pH 5.5) with 10% (*v/v*) dimethyl sulfoxide (DMSO), a well-tolerated corneal-penetration enhancer [105]. Mice received topical L-DOPA treatment in the form of two 10 μL eye drops in the left eye and an equal volume of vehicle solution (1× PBS, pH 5.5 with 10% DMSO) in the right eye twice daily (at 7:30 a.m. and 7:00 p.m.) for a period of six days starting from day 9 after immunization. The last dose of L-DOPA eye drops or i.p. injection was administered at 7:30 a.m. on day 14 after immunization. The optomotor response test on day 14 was conducted 1 h after L-DOPA or vehicle injection. The animals were then examined for fundus changes and immediately sacrificed to collect neuroretinas for flow cytometry analysis.

### 4.5. Optomotor Response

The optomotor response test was used to analyze visual acuity by monitoring the reflex response of mice to rotating high-contrast vertical stripes, which is manifested by movements of the head and/or body in the same direction as the rotation of the stripes. The optomotor apparatus (Figure S1a) was constructed based on the small optomotor drum design described by Schmucker et al. [40]. Briefly, the apparatus was composed of (1) a circular drum (diameter = 22 cm, height = 30 cm) on which the stimuli were displayed—vertical black and white stripes with a spatial frequency of 0.2 cycles/degree, (2) a rotating platform for the circular drum and a motor with a control unit controlling the rotation speed of the drum (50 degrees/s) and alternating direction of rotation (the drum rotated clockwise for 20 s and then counterclockwise for 20 s), (3) a circular stationary central platform (in height of 2 cm from the bottom of the circular drum) on which a transparent acrylic cylinder (diameter = 15 cm, height = 25 cm) for free movement of the mouse was placed, and (4) a digital RGB video camera (1920 × 1080 pixel resolution) for the monitoring of mouse movement on the frontal plane. A video camera was inserted into the cover of the transparent cylinder to a height of about 20 cm above the mouse. A total of 3000 K white LEDs (SMD2835, OptoFlash OPWW2835-6012EG) covering the full visible light spectrum mounted on the underside of the central platform (6 pieces, diameter = 70 mm) and on the cover of the transparent cylinder (12 pieces, diameter = 70 mm) were used as a light source. The light intensity of the LEDs was set to reach 30–35 cd/m^2^ in the center of the central platform at mouse eye height. Image processing and measurement of the mouse’s angular movements were adopted from Schmucker et al. [40] with some modifications. Before the measurement, the mice were allowed at least 10 min to acclimate to the optomotor arena. Once the mouse adapted to this arena and followed the rotation of the black and white stripes by moving its head and body, the video recording was switched on. Digital video images of the mice (Figure S1b) were collected at 30 frames/s. A script for processing the recorded videos was implemented in the Matlab version 2020b (MathWorks, Natick, MA, USA) environment. The total length of each processed recording was 480 s. As the rotation of the cylinder was controlled autonomously, it was necessary to detect the direction of rotation from the video record. This was implemented by selecting a cut of the video record in which two stripes were ideally visible, namely, the area directly above the center of the monitored area. This cutout was subsequently converted to a black and white (B/W) raster using adaptive thresholding. The rotation direction was determined by the order of the stripes and by comparing the B/W pixel counts with the previous frame. For each individual frame, two parameters were specified for the mouse—its orientation and its position in the cylinder (Figure S1d). The position of the mouse was determined by the position angle defining its location relative to the center of the cylinder, where 0° was directly above the center of the frame and 90° was the position to the right of the mentioned center. The orientation of the mouse was defined such that 0° was the vertical and 90° was the horizontal orientation in a frame (Figure S1d). The procedure for determining the above parameters was as follows: (1) The image was converted into grayscale; (2) the area in which the mouse was monitored was selected using a circular mask (Figure S1c); (3) the monitored area was converted into a B/W raster using a threshold value, which could be constant due to the exact lighting conditions, and consequently inverted, resulting in the mouse being marked with white pixels in the image (Figure S1d); (4) the mouse position angle was determined by the vertical axis of the image and the line joining the center of the monitored area and the mean of the white pixel positions; (5) the mouse orientation was determined by the angle between the vertical axis of the image and the regression line defined by the white pixels; (6) based on the frame rate, the angular velocities of the mouse position angle and orientation angle were determined; and (7) the mean values of the angular velocities of the mouse’s snout–tail axis (angular orientation speed) and of the center of the mouse’s body mass in relation to the center of the cylinder (angular running speed), at which the mouse was moving in the same direction as the cylinder (either clockwise or counterclockwise), were computed and used further for the statistical analysis.

### 4.6. Eye Fundus Imaging and EAU Clinical Score

Eye fundus images were taken using the topical endoscopy fundus imaging (TEFI) system described previously [21]. In brief, the mice were anesthetized with ketamine (100 mg/kg) and xylazine (10 mg/kg) and the pupils were dilated with tropicamide eye drops (1% Unitropic; Unimed Pharma, Bratislava, Slovakia). The fundus was examined for inflammatory changes such as engorged blood vessels or the presence of inflammatory infiltrates, linear lesions, papilledema, retinal hemorrhage, and retinal detachment. The severity of inflammation was evaluated separately for the optic disc, retinal vessels, and retinal tissue using EAU grading criteria in C57BL/6 mice, as described by Xu et al. [28]. The overall final inflammation grade of the retina was then calculated as the average score of these three components: (optic disc points + retinal vessel points + retinal tissue points)/3. After fundoscopy, an additional dose of ketamine and xylazine was administered to the EAU mice. The deeply anesthetized mice were then transcardially perfused with 20 mL of 1× PBS. The eyes and spleens were harvested and transferred to ice-cold RPMI-1640 with 10% FBS and processed for flow cytometry.

### 4.7. Flow Cytometry

The neuroretinas were excised from the eyes using a binocular stereomicroscope. First, the eyeball was punctured in the anterior part of the pars plana with a 30 G needle, and microdissecting scissors were inserted into the puncture to cut off the entire anterior portion of the eye. Next, the lens was pushed out and the neuroretina was lifted from the optic nerve head, pinched off at the point where it connects to the optic nerve, and gently pulled away from the eye cup with forceps. The vitreous humor, ora serrata, and remnants of the retinal pigment epithelium were separated from the neuroretina and discarded. The neuroretinas were dissociated into single cells by gentle pipetting in 3 mL of RPMI-1640 containing 10% FBS followed by collagenase D (1 mg/mL; Roche Diagnostics, Mannheim, Germany) digestion at 37 °C for 1 h and filtration through a 100 μm nylon cell strainer. Splenocyte cell suspension was prepared by mechanical dissociation using scissors, cutting the spleen into small fragments and gently pressing them through a 100 μm nylon cell strainer. The cell suspension was then depleted of red blood cells by hypotonic lysis with ammonium chloride and washed with RPMI-1640. For flow cytometry analysis of neuroretina cells and splenocytes, the cells were first incubated with anti-mouse CD16/CD32 antibody (60161; clone 2.4G2; dilution: 1/200; Stemcell Technologies, Vancouver, BC, Canada) to block Fc receptors. Neuroretina cells were then stained with the following anti-mouse monoclonal antibodies by eBioscience (Thermo Fisher Scientific, Waltham, MA, USA): eFluor 450-CD45.2 (48-0454-82; clone 104), eFluor 506-CD3 (69-0032-82; clone 17A2), Super Bright 702-CD4 (67-0042-82; clone RM4-5), FITC-CD19 (11-0193-82; clone eBio1D3 (1D3)), FITC-CD11c (11-0114-82; clone N418), PE-CD11b (12-0112-82; clone M1/70), and APC-CD8a (17-0081-82; clone 53-6.7). For splenocyte staining, the following anti-mouse monoclonal antibodies by eBioscience were used: eFluor 506-CD3 (69-0032-82; clone 17A2), Super Bright 702-CD4 (67-0042-82; clone RM4-5), FITC-CD11c (17-0114-82; clone N418), FITC-MHC Class II (I-A/I-E) (11-5321-82; clone M5/114.15.2), APC-CD44 (17-0441-82; clone IM7), eFluor 450-CD45R (B220) (48-0452-82; clone RA3-6B2), and Super Bright 702-CD11b (67-0112-82; clone M1/70). A fixable viability dye eFluor 780 (65-0865-18; eBioscience) was also included in staining panels for neuroretina cells and splenocytes to enable the exclusion of dead cells from the analysis. Flow cytometry data were acquired on an Attune NxT Flow Cytometer (Thermo Fisher Scientific, Waltham, MA, USA) and analyzed using Attune NxT version 3.1.2 software (Thermo Fisher Scientific, Waltham, MA, USA).

### 4.8. RNA Isolation and Quantitative Real-Time PCR (qRT-PCR)

The mice were dark adapted overnight and placed under a dim red light for all further procedures. The mice were deeply anaesthetized with an i.p. injection of ketamine (150 mg/kg) and xylazine (15 mg/kg), followed by cardiac perfusion with 20 mL of 1× PBS. The spleens and livers were excised, snap-frozen on dry ice, and stored at −80 °C until processed for RNA extraction. The eyes were carefully removed and immersed in saline (0.9% sodium chloride) in a Petri dish placed on ice. The neuroretinas were excised from the eyes in the dark using a binocular stereomicroscope with a far-red light source and the same technique used to collect the neuroretinas for flow cytometry. Total RNA was isolated from the mouse organs (neuroretina, spleen, and liver) and rat retinal capillary endothelial cells using the RNeasy Mini Kit (Qiagen, Hilden, Germany) according to the manufacturer’s recommendation with an on-column DNase digestion step using the RNase-Free DNase Set (Qiagen, Hilden, Germany) to eliminate genomic DNA. The obtained RNA was reversely transcribed to cDNA using the High-Capacity cDNA Reverse Transcription Kit (Applied Biosystems, Foster City, CA, USA). The qRT-PCR was performed using TaqMan Fast Advanced Master Mix (Applied Biosystems, Foster City, CA, USA) with TaqMan Gene Expression Assays (Applied Biosystems, Foster City, CA, USA) containing primer/probe sets for mouse (Mm) or rat (Rn) genes: *Adrb2* (Mm02524224_s1), *Aif1* (Mm00479862_g1), *Ccl2* (Mm00441242_m1 and Rn00580555_m1), *Ccl3* (Mm00441259_g1), *Ccl20* (Mm01268754_m1 and Rn01400117_g1), *Cd4* (Mm00442754_m1), *Crp* (Mm00432680_g1), *Cxcl1* (Mm04207460_m1), *Cxcl2* (Mm00436450_m1), *Cxcl10* (Mm00445235_m1 and Rn01413889_g1), *Drd4* (Mm00432893_m1), *Gfap* (Mm01253033_m1), *IL-1b* (Mm00434228_m1), *IL-6* (Mm00446190_m1 and Rn01410330_m1), *IL-17a* (Mm00439618_m1), and endogenous control glyceraldehyde-3-phosphate dehydrogenase (GAPDH; Mm99999915_g1 and Rn99999916_s1) according to the manufacturer’s instructions. The QuantStudio 7 Flex Real-Time PCR System (Applied Biosystems, Foster City, CA, USA) was used to carry out qRT-PCR. Relative mRNA expression of each gene was assessed using the relative standard curve method.

### 4.9. RNA Sequencing (RNA-seq) and Bioinformatics Analysis

The RNA isolated from dark-adapted neuroretinas (as described above) was used for RNA-seq. The quality of RNA was determined with the Agilent 2100 Bioanalyzer (Agilent Technologies, Santa Clara, CA, USA). Only samples with an RNA integrity value (RIN) ≥ 8.5 and a 28S/18S ratio ≥ 1.4 were used for library preparation. The sequencing libraries were prepared using the SMARTer Stranded Total RNA-Seq Kit v2 (Takara, Shiga, Japan) following the manufacturer’s instructions. The protocol involved the removal of ribosomal cDNA, without the loss of other cDNAs originating from non-coding or coding RNAs. Samples were sequenced with the NextSeq 500/550 High Output Kit v2 (75 cycles) and the NextSeq PhiX Control Kit (Illumina, San Diego, CA, USA) on a NextSeq 500 next-generation sequencer (Illumina) at the Genomics and Bioinformatics Core Facility, Institute of Molecular Genetics of the ASCR, Prague, Czech Republic.

For subsequent read processing, a bioinformatic pipeline nf-core/rnaseq version 1.4.2 [106] was used. Individual steps included removing sequencing adaptors and low-quality reads with Trim Galore! [107], mapping the data to reference genome GRCm38 and Ensembl annotation version 98 [108] with HISAT2 [109], and quantifying expression at the gene level with featureCounts [110]. Per-gene mapped counts served as input for differential expression analysis using the DESeq2 R Bioconductor package [111]. Prior to the analysis, genes not expressed in at least two samples were discarded. We supplied an experimental model assuming the sample group (*Gnat1^rd17^* vs. WT) as the main effect. The resulting per-gene expression log2-fold changes were used for differential expression analysis. Genes exhibiting a minimal absolute log2-fold change value of 1 and statistical significance (FDR 0.05) between compared groups of samples were considered differentially expressed. Next, gene-set overrepresentation analysis was done using a gene-length bias-aware algorithm implemented in the goseq R Bioconductor package [112] with KEGG (Kyoto Encyclopedia of Genes and Genomes) pathway gene sets. Enriched GO (Gene Ontology) terms of the protein-coding DEGs were identified with the MGI Gene Ontology Term Finder [113]. The raw sequencing data are available on the ArrayExpress database under accession number E-MTAB-10863.

### 4.10. Immunohistochemistry

The mice were dark adapted overnight prior to anesthesia and handled subsequently under dim red-light illumination throughout the subsequent procedures. After deep anesthesia with an i.p. injection of ketamine (150 mg/kg) and xylazine (15 mg/kg) and a cardiac perfusion with 20 mL of 1× PBS, the eyes were removed and freeze-embedded with a carboxymethyl cellulose gel SCEM (SECTION-LAB, Hiroshima, Japan) in cooled hexane. For immunohistochemistry, frozen blocks with embedded eyes were sectioned transversely at 10 µm thickness using a Leica CM3050 (Leica Microsystems, Wetzlar, Germany) cryostat microtome and an adhesive film (Cryofilm type IIIC (UF16); SECTION-LAB) as described by Kawamoto [114,115].

The eye sections attached to the adhesive film were dehydrated in 100% ethanol, fixed in 4% paraformaldehyde in 1× PBS for 15 min, and rinsed with 1× PBS. The sections were then blocked with 2% BSA in 1× Tris-buffered saline/0.1% Tween 20 (TBS-T) for 30 min at room temperature and incubated overnight at 4 °C with the following anti-mouse primary antibodies: rabbit anti-tyrosine hydroxylase (ab6211; dilution: 1/500; Abcam, Cambridge, UK), rabbit anti-DRD4 (ab135978; dilution: 1/250; Abcam, Cambridge, UK), rat anti-CD31 (550274; clone MEC 13.3; dilution: 1/50; BD Pharmingen, San Diego, CA, USA), rabbit anti-phospho (Ser276)-NF-κB p65 (SAB5700333; dilution: 1/100; Sigma-Aldrich, Darmstadt, Germany), rabbit anti-phospho-Stat3 (Tyr705) (D3A7) XP (9145; dilution: 1/200; Cell Signaling Technology, Danvers, MA, USA), biotin rat anti-CD4 (100508; clone RM4-5; dilution: 1/200; BioLegend, San Diego, CA, USA), and biotin rat anti-CD11b (101204; clone M1/70; dilution: 1/200; BioLegend). Additionally, negative control sections were incubated with appropriate isotype control antibodies to distinguish nonspecific background staining from antigen-specific antibody staining. For sections stained with biotinylated antibodies, the endogenous avidin and biotin binding sites were blocked with the Avidin/Biotin Blocking Kit (Vector Laboratories, Burlingame, CA, USA) following the manufacturer’s instructions. After washing twice in 1× PBS, sections were incubated with species-matched fluorochrome-conjugated secondary antibodies and with Hoechst 33342 fluorescent stain (H3570; dilution: 1/10,000 dilution; Thermo Fisher Scientific, Waltham, MA, USA) at room temperature for 1 h. The following secondary antibodies purchased from Thermo Fisher Scientific, Waltham, MA, USA, were used: donkey anti-rabbit IgG (H+L) highly cross-adsorbed, Alexa Fluor Plus 647 (A32795; dilution: 1/500); goat anti-rabbit IgG (H+L) highly cross-adsorbed, Alexa Fluor 488 (A11034; dilution: 1/500); goat anti-Rat IgG (H+L) cross-adsorbed, Alexa Fluor 647 (A-21247; dilution: 1/500); and Streptavidin, Alexa Fluor 488 conjugate (S32354; dilution: 1/500). The sections were visualized using an automated scanning microscope, ZEISS Axio Scan.Z1 (Carl Zeiss AG, Oberkochen, Germany), and the acquired images were analyzed and adjusted for contrast and brightness using the Zen version 2.1 (blue edition) software (Carl Zeiss AG, Oberkochen, Germany). The Zen version 2.1 software was also used to measure ONL and INL thickness in the central (at 500 μm from either side of the optic nerve head) and peripheral retina (at 500 μm from each margin of the ciliary body).

### 4.11. Western Blot and ELISA Analyses

After overnight dark adaptation, the mice were placed under dim red-light illumination, which was kept on throughout the subsequent procedures, including neuroretina isolation. The mice were deeply anaesthetized with an i.p. injection of chloral hydrate (400 mg/kg). Blood was collected by cardiac puncture to obtain serum samples for CRP and catecholamine determination, and the mice were then transcardially perfused with 20 mL of 1× PBS. The eyes were removed and the neuroretinas were isolated using the same technique as described for neuroretina collection for flow cytometry. Each neuroretina was immersed in 160 μL of ice-cold 1× RIPA buffer (Abcam, Cambridge, UK) containing Protease and Phosphatase Inhibitor Cocktail (Sigma-Aldrich, Darmstadt, Germany), 0.01 N HCl, 1 mM EDTA, and 4 mM sodium metabisulfite to prevent catecholamine degradation. Samples were then homogenized on ice at 5000 rpm with 15–20 pestle strokes using a glass Potter–Elvehjem tissue grinder with a PTFE pestle attached to an overhead stirrer (Wheaton, DWK Life Sciences, Millville, NJ, USA). Homogenates were transferred to microcentrifuge tubes and ultrasonic pretreatment was carried out for 5 min (30 s on/off per minute cycle) at 4 °C using the Q700 Sonicator (QSonica LLC, Newtown, CT, USA) coupled with a cup horn. Following that, homogenates were clarified at 15,000× *g* for 10 min at 4 °C, and the supernatant was aliquoted and assayed for total protein concentration using the BCA protein assay kit (Sigma-Aldrich, Darmstadt, Germany), Western blot, and ELISA analyses. 

For Western blot analysis, the aliquots of neuroretina homogenates and cytoplasmic and nuclear extracts of rat retinal capillary endothelial cells were diluted to an equal protein amount using Protein Sample Loading Buffer (LI-COR Biosciences, Lincoln, NE, USA) containing dithiothreitol (DTT) and heated for 5 min at 95 °C. The samples and protein ladder (Precision Plus Protein Dual Color Standards; Bio-Rad Laboratories, Hercules, CA, USA) were loaded onto commercially prepared 10% Mini-PROTEAN TGX Stain-FREE precast gels (Bio-Rad Laboratories, Hercules, CA, USA) and subjected to electrophoresis separation, followed by electrophoretic transfer to a polyvinylidene difluoride membrane (Immun-Blot PVDF Membrane; Bio-Rad Laboratories, Hercules, CA, USA). The quality of the protein transfer was checked by Ponceau S staining (Sigma-Aldrich, Darmstadt, Germany). Membranes were blocked using Every Blot Blocking Buffer (Bio-Rad Laboratories, Hercules, CA, USA) according to the manufacturer’s instructions and incubated overnight at 4 °C with the following rabbit anti-mouse and anti-rat primary antibodies (all with dilution 1/1000): anti-Gnat1 (GTX105960-100; GeneTex, Irvine, CA, USA), anti-DRD4 (ab135978; Abcam, Cambridge, UK), anti-β2-AR (ab182136; Abcam, Cambridge, UK), anti-phospho (Ser276)-NF-κB p65 (3034; Cell Signaling Technology), anti-phospho (Tyr705)-Stat3 (D3A7) XP (9145; Cell Signaling Technology), anti-histone H3 (ab1791; Abcam, Cambridge, UK), anti-GAPDH (PA1-987; Themo Fisher Scientific), and anti-vinculin (13901; Cell Signaling Technology). The following day, the membranes were washed and incubated with goat anti-rabbit secondary antibody conjugated with horseradish peroxidase (ab97051; dilution: 1/10,000; Abcam, Cambridge, UK). Chemiluminescence was achieved by applying Radiance Plus Substrate with Radiance Peroxide (AC2103; Azure Biosystems, Dublin, CA, USA) to the membranes. The developed chemiluminescence was captured using the Azure c300 Digital Imager (Azure Biosystems, Dublin, CA, USA) and the detected band densities were quantified using AzureSpot version 14.2 software (Azure Biosystems, Dublin, CA, USA). 

Concentrations of DA, NE, and EPI in the neuroretina homogenates and serum were determined using the 3-CAT Research ELISA Kit (BA E-5600R; Labor Diagnostika Nord, Nordhorn, Germany), and CRP serum levels were assessed using the mouse CRP ELISA Kit (EM20RB; Thermo Fisher Scientific, Waltham, MA, USA). Protocols provided by the manufacturers were followed for each ELISA kit. Absorbance was measured at 450 nm using a microplate reader, BioTek 800 TS (BioTek, Winooski, VT, USA), and data were analyzed with Gen5 version 3.0 software (BioTek, Winooski, VT, USA). Concentrations of neuroretinal catecholamines were normalized to 1 mg of total protein content in the homogenate. 

### 4.12. Statistical Analysis

GraphPad InStat version 3.1 and Prism version 8 software (GraphPad, San Diego, CA, USA) were used to conduct statistical analysis. Comparisons between two groups were performed using the unpaired *t*-test for unpaired data or the paired *t*-test for paired data. Differences among multiple groups were tested by one-way analysis of variance (ANOVA) followed by the Tukey–Kramer multiple comparison test. Statistical significance was determined at a *p*-value of less than 0.05.

## Data Availability

All data generated during this study are included in this paper or the supplementary materials. The raw sequencing data are available on the ArrayExpress database under accession number E-MTAB-10863.

## Supplementary Materials

The following are available online at https://www.mdpi.com/article/10.3390/ijms23010453/s1.
