# Supplementary material for: Depletion of Retinal Dopaminergic Activity in a Mouse Model of Rod Dysfunction Exacerbates Experimental Autoimmune Uveoretinitis: A Role for the Gateway Reflex
Source: Int J Mol Sci. 2021 Dec 31;23(1):453. doi: 10.3390/ijms23010453 (PMC8745287; doi:10.3390/ijms23010453)
Supplement: Supplementary file 1 [file ijms-23-00453-s001.zip › FIgures S1-S5.pdf]

Figure S1

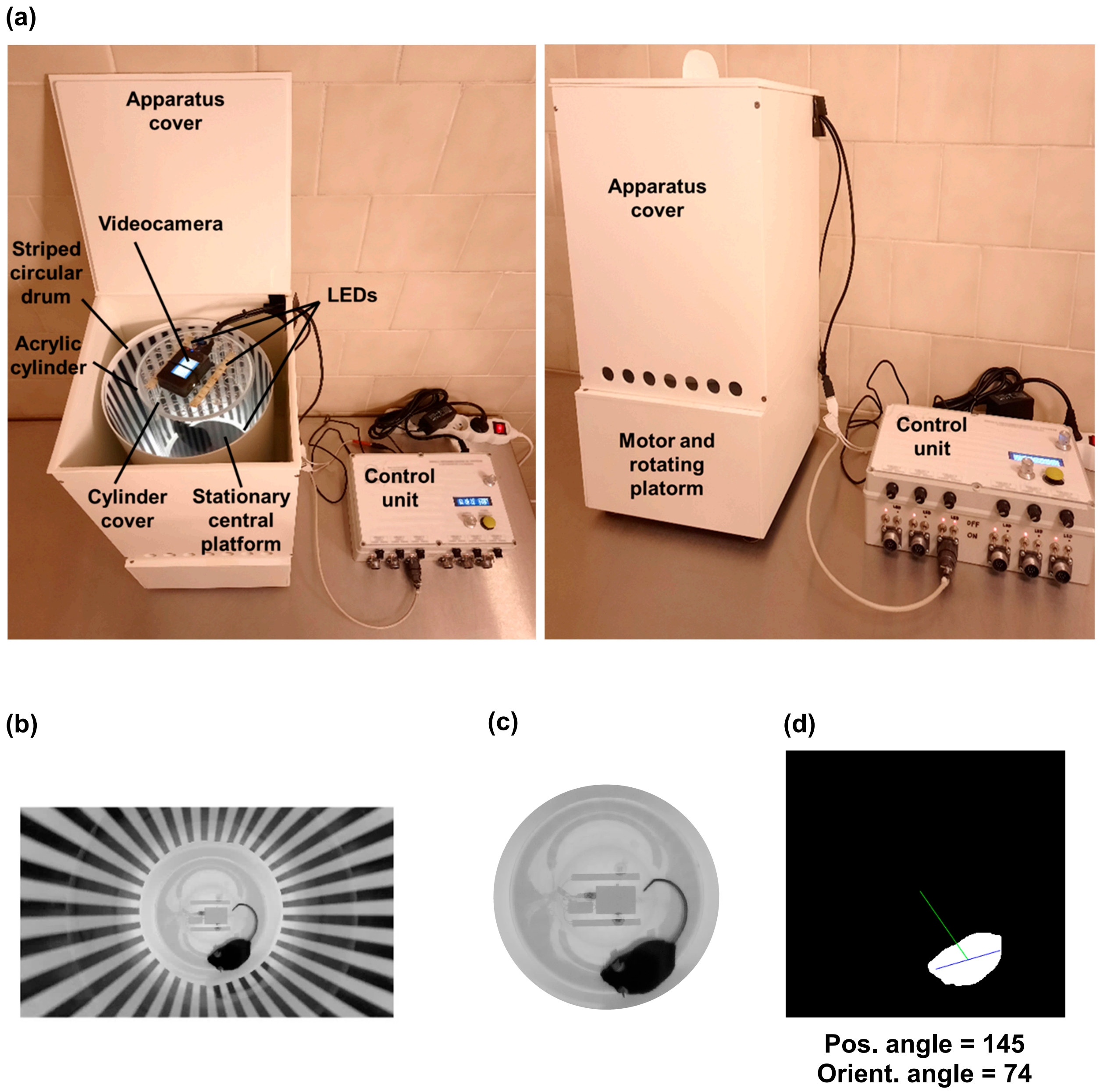

Figure S1. (a) The optomotor apparatus and an illustration of image processing for optomotor response analysis: **(b)** an input image, **(c)** an area in which the mouse is being tracked, and **(d)** the detected mouse with corresponding angles.

Figure S2

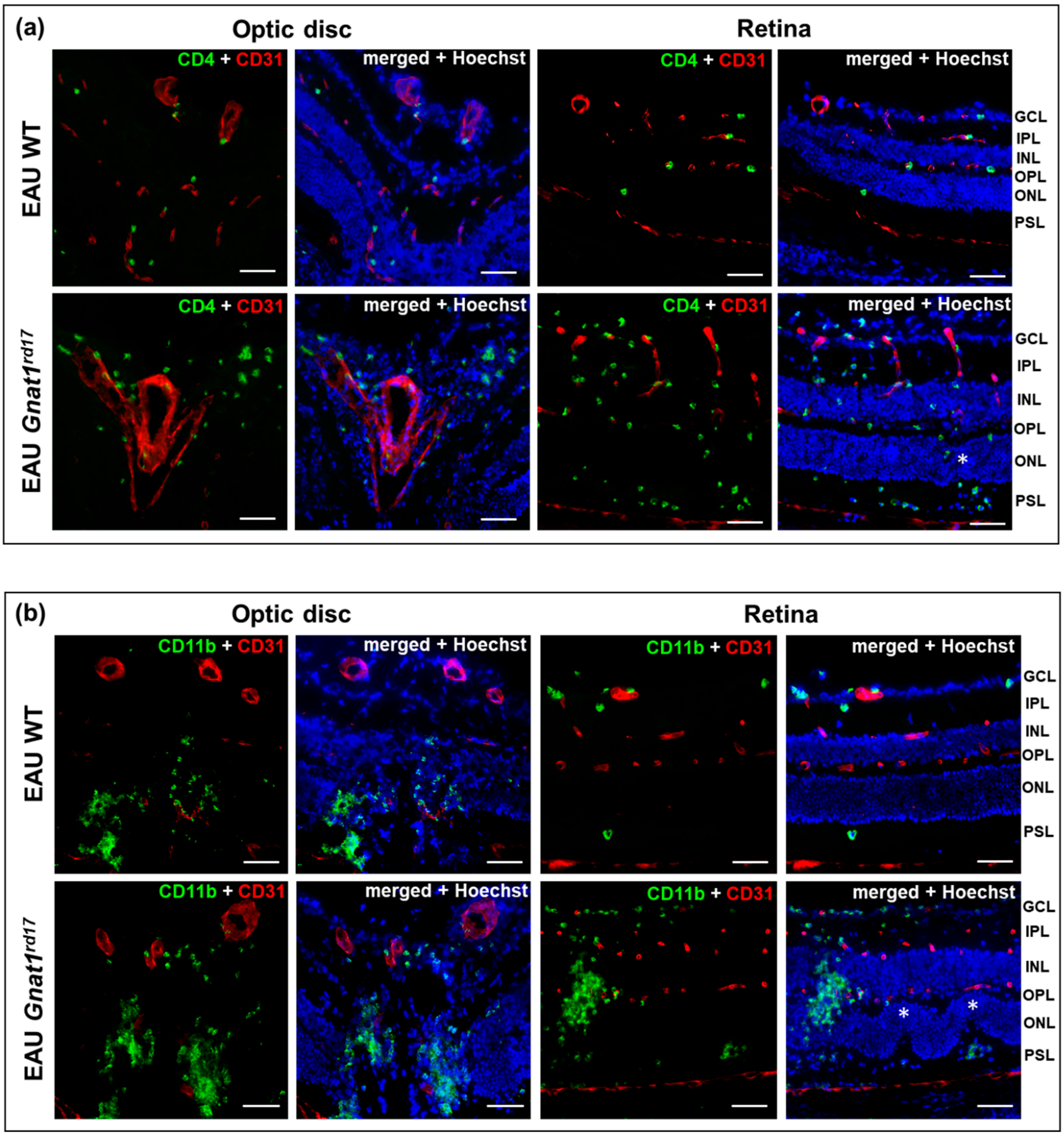

Figure S2. Immunofluorescence staining of infiltrated **(a)** $CD4^+$ T cells and **(b)** $CD11b^+$ cells around retinal vessels in the optic disc and retina. Representative retinal transverse sections of EAU WT and *Gnat1^{rd17}* mice (n = 5 animals per each group) on day 14 after immunization. Red represents CD31-immunoreactivity (CD31-ir; a marker of endothelial cells); green represents CD4-ir and CD11b-ir; blue represents nuclear stain Hoechst; and an asterisk represents the retinal fold caused by retinal detachment. The CD4-ir in EAU WT mice is shown in the very peripheral retina, as the CD4-ir was only sparsely found in the central retina. GCL, ganglion cell layer; IPL, inner plexiform layer; INL, inner nuclear layer; OPL, outer plexiform layer; ONL, outer nuclear layer; PSL, photoreceptor segment layer. The scale bar is 50 μm.

Figure S3

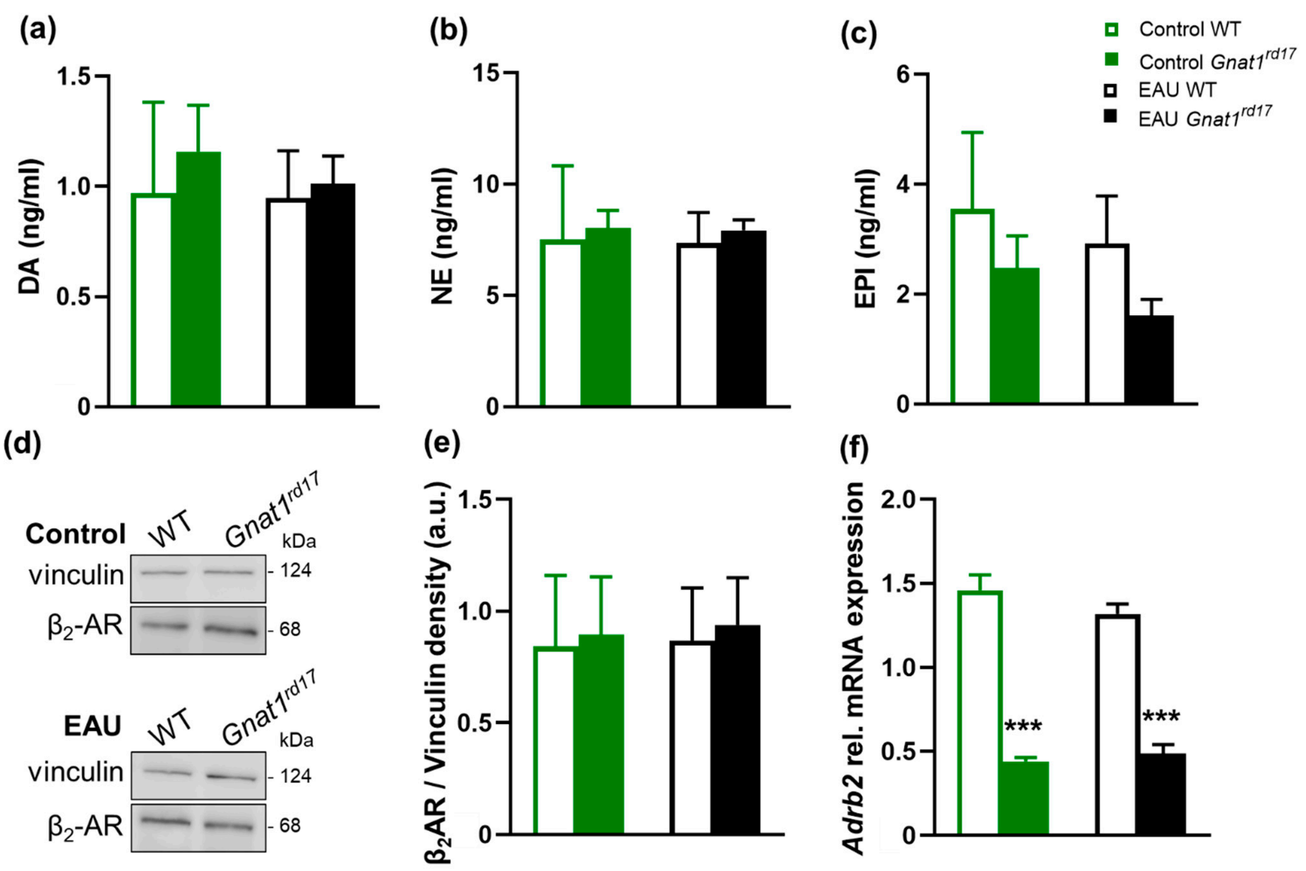

Figure S3. Serum catecholamine levels and retinal β_2-AR expression in *Gnat1^{rd17}* mice. Serum **(a)** DA, **(b)** NE, and **(c)** EPI levels in WT and *Gnat1^{rd17}* with and without EAU on day 14 after immunization. The results are presented as the mean ± SEM of 5 animals per group and were analyzed by one-way ANOVA followed by the Tukey-Kramer multiple comparison test. In two independent experiments, no significant differences were found between groups. **(d)** Representative western blot images and **(e)** relative protein expressions of β_2-AR determined by densitometry and normalized to vinculin as a loading control. Data are presented as the mean ± SEM of 5 to 6 neuroretinas from five control and six EAU mice on day 14 after immunization, both WT and *Gnat1^{rd17}*. Results are representative of two independent experiments and were analyzed by an unpaired *t*-test. **(f)** qRT-PCR results of mRNA expression for β_2-AR in the neuroretinas of control and EAU mice on day 14 after immunization, both WT and *Gnat1^{rd17}*. Data are presented as the mean ± SEM of 10 neuroretinas from 5 mice pre group. The unpaired t-test yielded a significant difference (***$P < 0.001$) between WT and *Gnat1^{rd17}*groups.

Figure S4

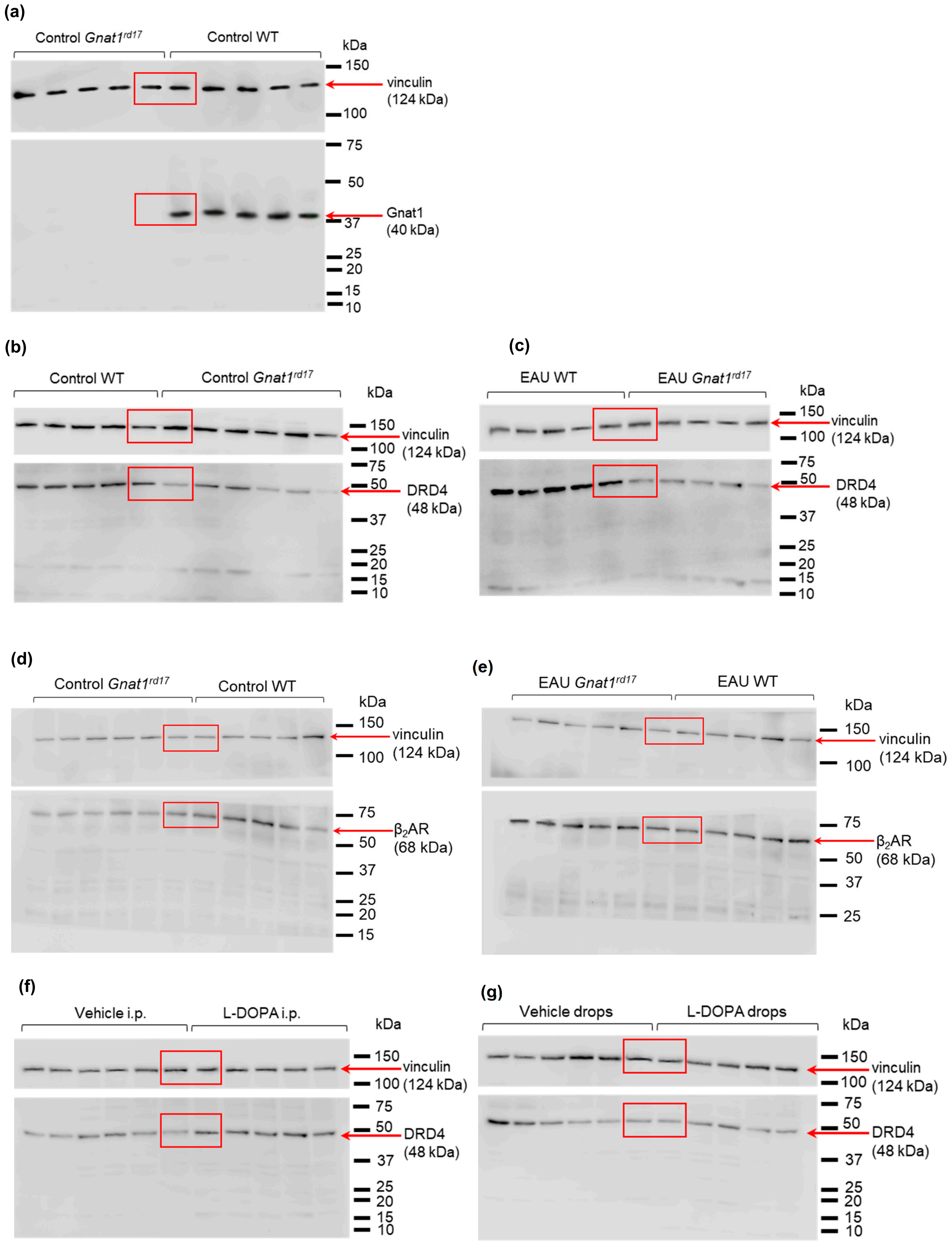

(a)
Control Gnat1rd17
Control WT
kDa
150
100
75
50
37
25
20
15
10
vinculin (124 kDa)
Gnat1 (40 kDa)
(b)
Control WT
Control Gnat1rd17
vinculin (124 kDa)
DRD4 (48 kDa)
(c)
EAU WT
EAU Gnat1rd17
vinculin (124 kDa)
DRD4 (48 kDa)
(d)
Control Gnat1rd17
Control WT
vinculin (124 kDa)
β2AR (68 kDa)
(e)
EAU Gnat1rd17
EAU WT
vinculin (124 kDa)
β2AR (68 kDa)
(f)
Vehicle i.p.
L-DOPA i.p.
vinculin (124 kDa)
DRD4 (48 kDa)
(g)
Vehicle drops
L-DOPA drops
vinculin (124 kDa)
DRD4 (48 kDa)

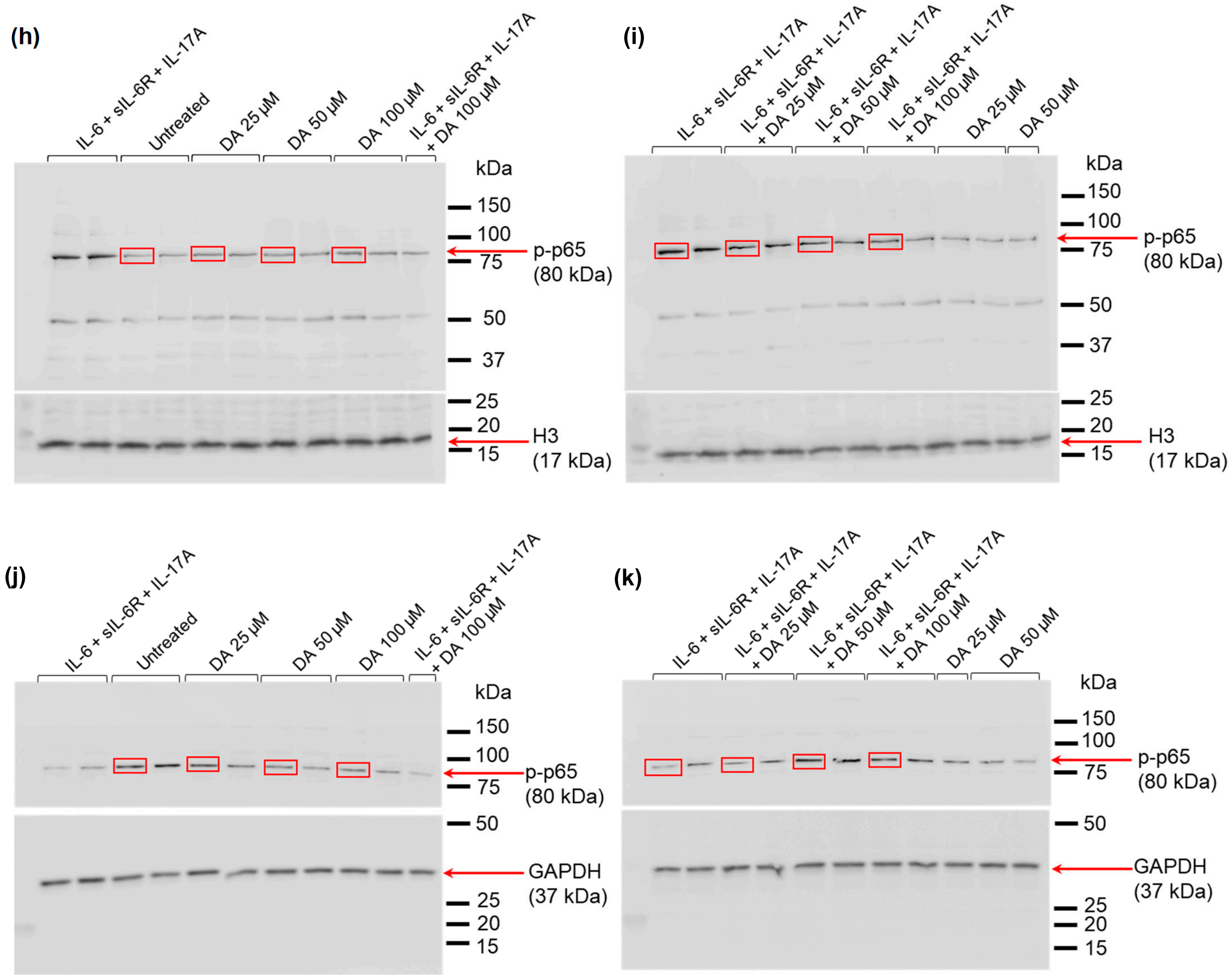
(h)
IL-6 + sIL-6R + IL-17A
Untreated
DA 25 µM
DA 50 µM
DA 100 µM
IL-6 + sIL-6R + IL-17A + DA 100 µM
kDa
150
100
75
50
37
25
20
15
p-p65 (80 kDa)
H3 (17 kDa)
(i)
IL-6 + sIL-6R + IL-17A
IL-6 + sIL-6R + IL-17A + DA 25 µM
IL-6 + sIL-6R + IL-17A + DA 50 µM
IL-6 + sIL-6R + IL-17A + DA 100 µM
DA 25 µM
DA 50 µM
kDa
150
100
75
50
37
25
20
15
p-p65 (80 kDa)
H3 (17 kDa)
(j)
IL-6 + sIL-6R + IL-17A
Untreated
DA 25 µM
DA 50 µM
DA 100 µM
IL-6 + sIL-6R + IL-17A + DA 100 µM
kDa
150
100
75
50
25
20
15
p-p65 (80 kDa)
GAPDH (37 kDa)
(k)
IL-6 + sIL-6R + IL-17A
IL-6 + sIL-6R + IL-17A + DA 25 µM
IL-6 + sIL-6R + IL-17A + DA 50 µM
IL-6 + sIL-6R + IL-17A + DA 100 µM
DA 25 µM
DA 50 µM
kDa
150
100
75
50
25
20
15
p-p65 (80 kDa)
GAPDH (37 kDa)

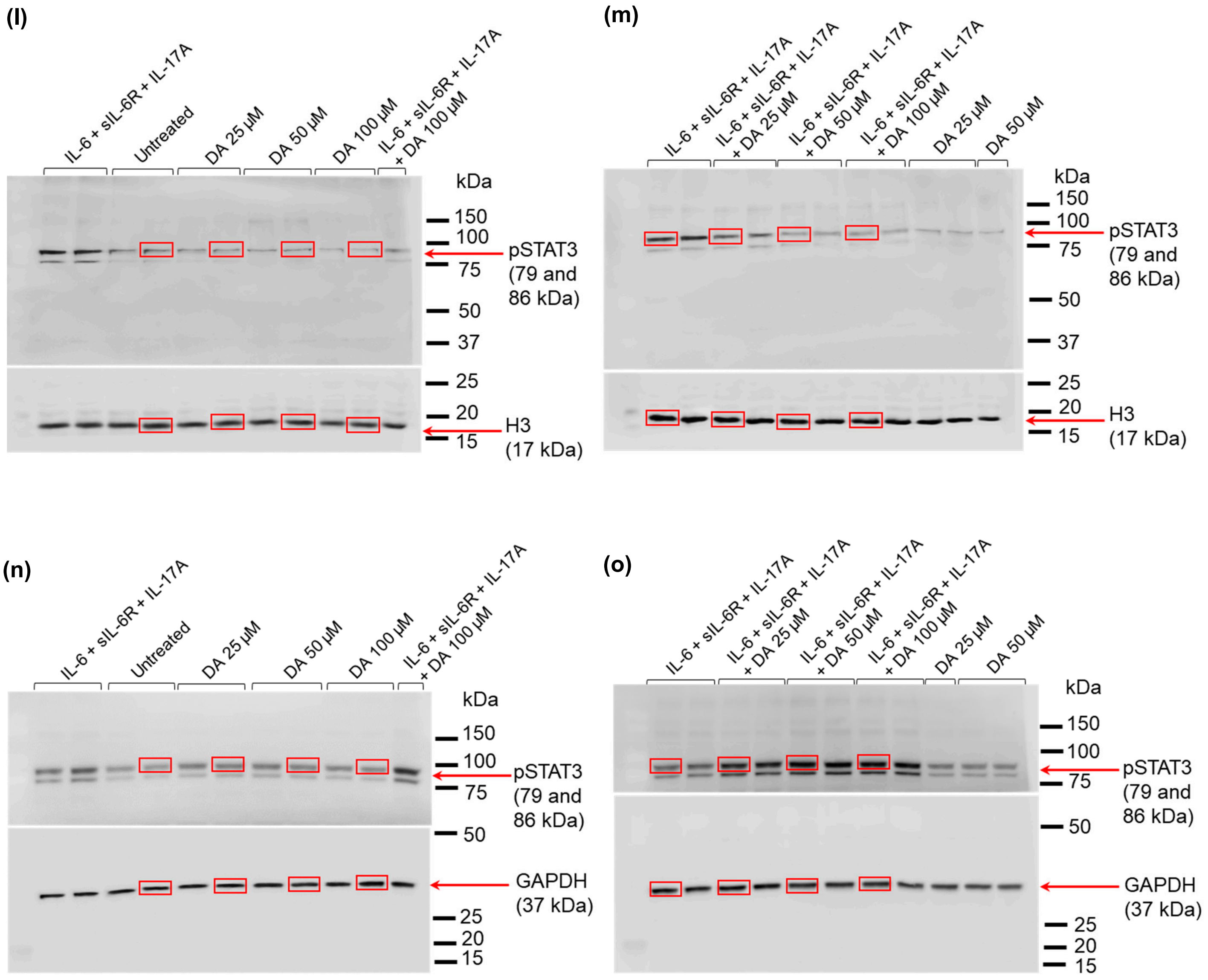

Figure S4. Complete western blot membrane images of Gnat1, DRD4, β_2-AR, p-pSTAT3, p-p65, and respective protein loading controls (vinculin, H3, and GAPDH). Red boxes mark the cropped images corresponding to Figures 3, 5, 9, 11, and S3. The membranes were cut based on molecular weights and were probed separately with antibodies for the proteins of interest and the protein loading controls.

Figure S5

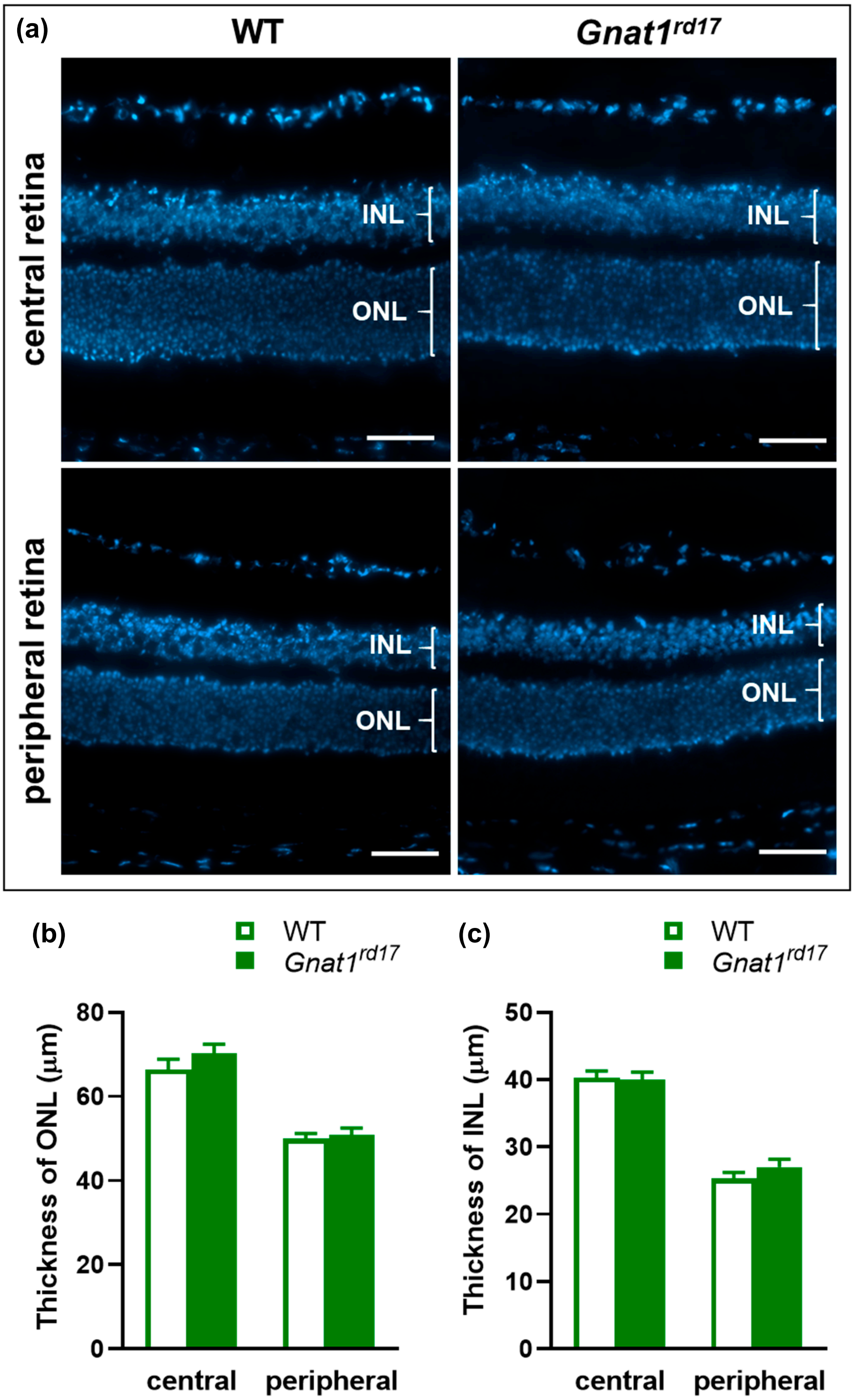

Figure S5. Analysis of retinal thickness in 8– to 10–week-old WT and *Gnat1*^{rd17} mice. **(a)** Representative images of Hoechst staining in the central and peripheral retinas and **(b)** quantification of the thicknesses of the inner nuclear layer (INL) and outer nuclear layer (ONL). Data are expressed as mean ± SEM, where n = 10 mice for each group. Unpaired *t*-test showed no significant differences between groups. The scale bar is 50 μm.
